# Foraging decisions with conservation consequences: Interaction between beavers and invasive tree species

**DOI:** 10.1002/ece3.8899

**Published:** 2022-05-15

**Authors:** Erika Juhász, Ákos Bede‐Fazekas, Krisztián Katona, Zsolt Molnár, Marianna Biró

**Affiliations:** ^1^ Department of Plant Systematics, Ecology and Theoretical Biology Institute of Biology Eötvös Loránd University Budapest Hungary; ^2^ Centre for Ecological Research Institute of Ecology and Botany Vácrátót Hungary; ^3^ Centre for Ecological Research GINOP Sustainable Ecosystems Group Tihany Hungary; ^4^ Faculty of Science Department of Environmental and Landscape Geography Eötvös Loránd University Budapest Hungary; ^5^ 72402 Department of Wildlife Biology and Management Institute for Wildlife Management and Nature Conservation Hungarian University of Agriculture and Life Sciences Gödöllő Hungary

**Keywords:** alluvial forest, *Castor fiber*, central‐place foraging strategy, ecosystem engineer species, floodplain, invasion ecology, optimal foraging strategy

## Abstract

1. Herbivore species can either hinder or accelerate the invasion of woody species through selective utilization. Therefore, an exploration of foraging decisions can contribute to the understanding and forecasting of woody plant invasions. Despite the large distribution range and rapidly growing abundance of beaver species across the Northern Hemisphere, only a few studies focus on the interaction between beavers and invasive woody plants.
2. We collected data on the woody plant supply and utilization at 20 study sites in Hungary, at two fixed distances from the water. The following parameters were registered: taxon, trunk diameter, type of utilization, and carving depth. Altogether 5401 units (trunks and thick branches) were identified individually. We developed a statistical protocol that uses a dual approach, combining whole‐database and transect‐level analyses to examine foraging strategy.
3. Taxon, diameter, and distance from water all had a significant effect on foraging decisions. The order of preference for the four most abundant taxa was *Populus* spp. (softwood), *Salix* spp. (softwood), *Fraxinus pennsylvanica* (invasive hardwood), and *Acer negundo* (invasive hardwood). The diameter influenced the type of utilization, as units with greater diameter were rather carved or debarked than felled. According to the central‐place foraging strategy, the intensity of the foraging decreased with the distance from the water, while both the taxon and diameter selectivity increased. This suggests stronger modification of the woody vegetation directly along the waterbank, together with a weaker impact further from the water.
4. In contrast to invasive trees, for which utilization occurred almost exclusively in the smallest diameter class, even the largest softwood trees were utilized by means of carving and debarking. This may lead to the gradual loss of softwoods or the transformation of them into shrubby forms. After the return of the beaver, mature stages of softwood stands and thus the structural heterogeneity of floodplain woody vegetation could be supported by the maintenance of sufficiently large active floodplains.
5. The beaver accelerates the shift of the canopy layer's species composition toward invasive hardwood species, supporting the enemy release hypothesis. However, the long‐term impact will also depend on how plants respond to different types of utilization and on their ability to regenerate, which are still unexplored issues in this environment. Our results should be integrated with knowledge about factors influencing the competitiveness of the studied native and invasive woody species to support floodplain conservation and reconstruction.

## Graphical abstract

A complex protocol was applied for the exploration of foraging decisions made by an ecosystem engineer species in an environment transformed by invasive tree species. The significant effects of the taxon, trunk diameter, and distance from water on foraging decisions, as well as the statements of central place foraging strategy were proven. Beavers might enhance the spread of invasive species because of the preferential utilization of softwoods, while they also influence the structure of floodplain vegetation.

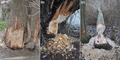

## 1 INTRODUCTION

### 1.1 Foraging strategy and the impact of herbivory

According to the optimal foraging strategy, mammalian herbivores select their dietary components under pressure from several constraints, including digestive capacity and morphophysiology, energy and nutrient requirements, as well as searching and handling time (Belovsky, 1984, 1997; Hanley, 1982; Redjadj et al., 2014). Foraging decisions depend on the nutrient and secondary plant compound contents of their food plants, the structural plant defense mechanisms, the size of the available items, and required traveling distances (Belovsky & Schmitz, 1994; Champagne et al., 2020; Jenkins, 1980).

Foraging strategies are not only important from a general ecological point of view but may also have serious consequences for conservation. Selective foraging and other disturbances caused by herbivory can lead to divergent effects on the ecosystems, both flora (Reimoser & Putman, 2011) and fauna (Katona & Coetsee, 2019). Herbivore‐induced changes in the vegetation dynamics and composition can be beneficial or disadvantageous from a nature conservation perspective, as there are large differences in biodiversity impact across habitats and for different herbivore–plant interactions (Cook‐Patton et al., 2014; Hester et al., 2000; Olff & Ritchie, 1998; Schäfer et al., 2019).

One of the common unfavorable consequences of selective herbivory is the dominance of less consumed plants due to their unpalatability, toxic substance content, or physical defense mechanisms (Augustine & McNaughton, 1998). Native herbivorous mammals may show a stronger preference for native species than for non‐native, invasive ones (Averill et al., 2016). This can promote an increase in the abundance of some invasive plant species without adequate herbivory control (see enemy release hypothesis, Keane & Crawley, 2002).

However, herbivores can also mitigate the spread of invasive plants by intensively consuming them (Katona et al., 2013; Marty, 2005; Schindler et al., 2016), reducing the performance of early and adult life‐history stages of these species (Maron & Vilà, 2001). Analyzing this latter impact, the biotic resistance hypothesis, Levine et al. (2004) stated that in many of the reviewed studies, mammalian herbivory can reduce invader establishment or fecundity to zero. Another global meta‐analysis found that native herbivores decreased the relative abundance of exotic plants by 28%, while exotic herbivores increased it by 65% (Parker et al., 2006). A deeper understanding of herbivory can help to improve the applied conservation management practices in natural habitats, for example, in wetlands (Biró et al., 2020; Molnár et al., 2020).

### 1.2 Foraging strategy of beavers and its impact in the light of the biological invasion

**FIGURE 1:**
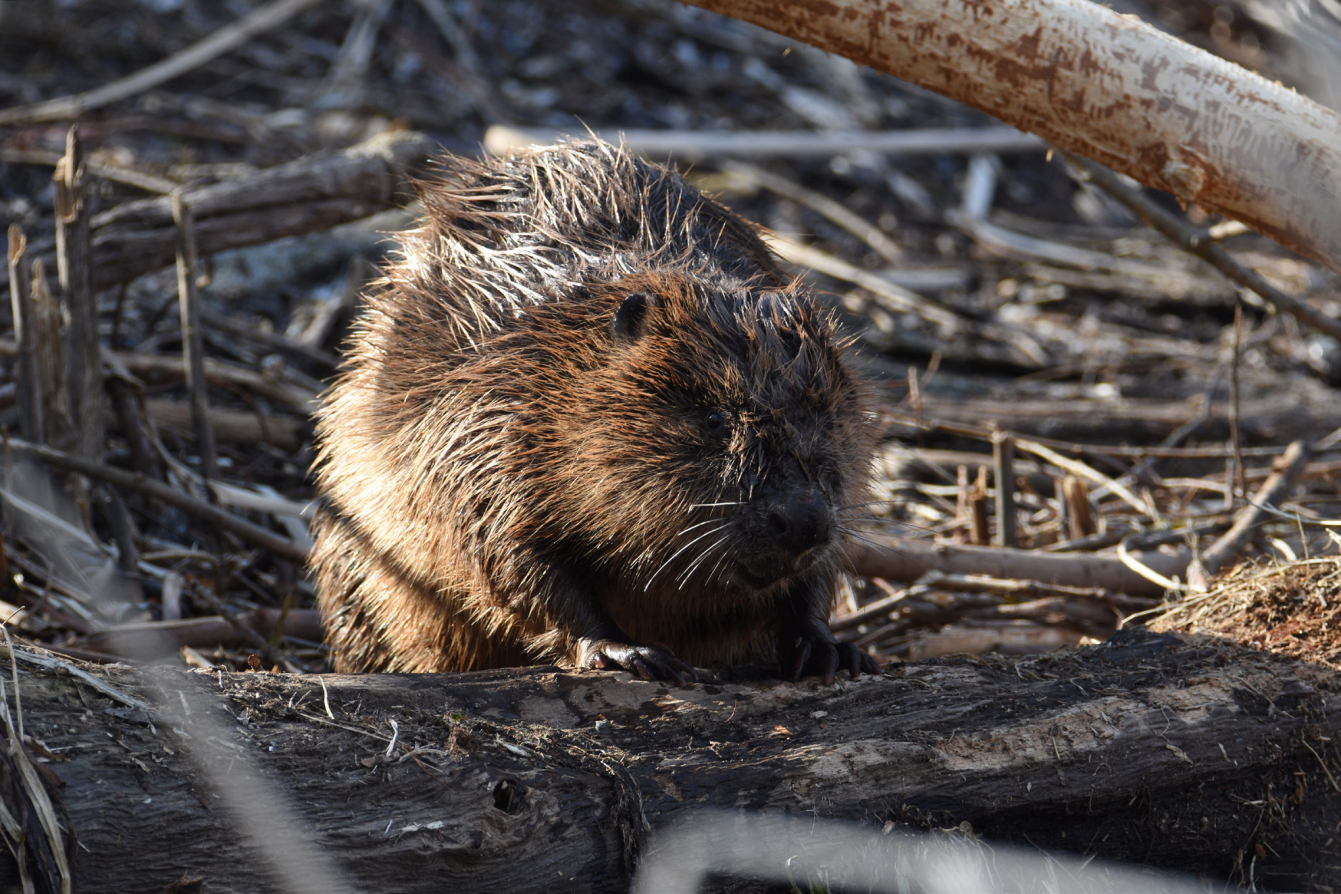
Eurasian beaver (*Castor fiber*). Photo: Juhász, E

In prehistoric times, the beaver (*Castor*) genus occupied all the cold and temperate climatic regions of the Northern Hemisphere, playing a key role in shaping the wetland habitats as ecosystem engineers (Halley & Rosell, 2002; Naiman et al., 1988). Both the Eurasian beaver (*Castor fiber*) (Figure 1) and the North American beaver (*Castor canadensis*) were subjected to intense hunting, which drastically reduced the population of the species by the middle of the 19th century (Nolet & Rosell, 1998; Wohl, 2019). As a result of reintroductions and conservation efforts, they are now widespread again, and by the latest estimation, the world population of the Eurasian beaver now exceeds 1.4 million (Halley et al., 2021).

Beavers (*Castor* spp.), as model animals, play an important role in the research of foraging strategies (Belovsky, 1984; Fryxell & Doucet, 1993; Gallant et al., 2016; Salandre et al., 2017). They are central place foragers, which means that the animals search for food items at various distances starting from a fixed location (Basey et al., 1988; Fryxell & Doucet, 1991). The key elements of beavers’ forage selection on woody plants are species preference, diameter selectivity, and distance from the water (Gallant et al., 2004; Haarberg & Rosell, 2006; Jenkins, 1980).

The spectrum of woody species on which beavers feed is wide‐ranging, although softwood species (*Salix* and *Populus* spp.) are usually preferred, as has been demonstrated in different habitats and in the presence of different foraging supplies for both the Eurasian beaver (Haarberg & Rosell, 2006; Vorel et al., 2015) and the North American beaver (Gallant et al., 2004; Gerwing et al., 2013; Salandre et al., 2017). However, in riparian woodlands of both Europe and North America, native softwood species are being replaced at a rapid rate by invasive woody species of other genera (Birken & Cooper, 2006; Saccone et al., 2010). Temperate and boreal softwood riparian woodlands are the most invaded woodland habitats in Europe (Wagner et al., 2017). Softwoods are foundation species of the floodplain vegetation, so supporting their survival and renewal is among the key objectives of river corridor reconstruction (Briggs & Osterkamp, 2021).

A few scientific papers have already documented the impact of the North American beaver on the invasion, in relation to its preferences and the responses of vegetation to the beaver‐made disturbance (North America: Lesica & Miles, 2004; Barela & Frey, 2016; South America: Rossell et al., 2014). Mortenson et al. (2008) also drew attention to the possible link between the abundance of invasive species and beaver activity during a spatial analysis. A potential conservation conflict was recently highlighted between the protection of the Eurasian beaver (EU Habitat Directive, Annex II and IV, EC, 1992) and the conservation of softwood gallery forests (EU Habitat Directive, Annex I, EC, 1992), arising from the unfavorable effects of selective foraging (Juhász et al., 2020). In this paper, we analyze in depth the beaver's foraging strategy in this environment. The complex assessment of the beaver's foraging decisions in the presence of invasive species is a novel field of research (Deardorff & Gorchov, 2021).

Beaver activity affects not only the proportion of species but also the structure of waterbank vegetation (Jones et al., 2009; Mahoney & Stella, 2020). Different diameter classes are often utilized at different ratios, and diameter selectivity may differ among taxa (Basey et al., 1988; Haarberg & Rosell, 2006; Jackowiak et al., 2020). Thus, the utilization of certain diameter classes of a given taxon may also be relevant to biological invasion, if the diameter‐class distribution of native and invasive species is not the same. The effects of beavers are more complex, due to the fact that not all of the utilized trees are felled, some are only debarked or carved. To the best of our knowledge, no scientific literature is available about the variation in the frequency of these utilization types by taxon and diameter category. However, the type and extent of wounds can affect the ability of trees to survive and regenerate (Delvaux et al., 2010; Vacek et al., 2020). Foraging intensity, as well as taxon and diameter selectivity, are also influenced by the distance from the water, according to the optimal and the central‐place foraging strategy (Jenkins, 1980), so the beaver impact varies on a small spatial scale.

A deep analysis of the factors behind selective utilization could provide information about the magnitude of the beavers’ effect on different taxa and diameter classes, and consequently about changes in the diameter class distribution and the alteration of the species’ frequency in the canopy layer. A detailed description of the impact of beavers would be an important step in understanding the invasion dynamics, which is essential knowledge for the development of future conservation management and restoration of active floodplains. Accordingly, in this paper, we examine the factors behind the foraging decisions of the Eurasian beaver at 20 study sites in Central European temperate floodplains in the Danube River Basin.

Our main objective was to answer the following questions:

1. Is taxon selectivity more important than other factors (diameter and distance from the water) in the foraging strategy of the beaver (*C*. *fiber*)?
2. To what extent does the beaver utilize softwood species and the most abundant invasive hardwood species?
3. Does diameter selectivity differ between softwoods and invasive hardwoods?
4. Is there a difference in the type of utilization (felling, carving, or debarking) between taxa and between trunk diameter categories?
5. Is there a difference in the beaver's forage selection directly along the waterbank and at a distance of 10 m from the waterbank?

We assume that H1: the beaver prefers softwoods (*Salix* and *Populus* spp.) to the most abundant invasive hardwood species in our region (*Acer negundo* and *Fraxinus pennsylvanica*) and H2: the utilization of larger trunk diameters is more typical for preferred softwood species than for invasive species. If these hypotheses are true, invasive species may gain a competitive advantage in the canopy layer, and their older specimens may be released from the effects of the beaver. Furthermore, we expect H3: a lower frequency of felling among non‐preferred taxa and in larger diameter classes, and H4: a stronger selectivity further from the water.

## 2 STUDY AREA AND METHODS

### 2.1 Study area and study sites

Our study was performed in Hungary, Central Europe, where rivers in the lowland landscape formed extensive floodplains in historical times, measuring up to several hundred thousand hectares in area (http1: http://www.kotivizig.hu/doksik/akk/mellekletek/2_melleklet/2_3_1_terkep.pdf). River regulations began in 1846, resulting in a radical decrease in the active floodplains and large‐scale transformations of the whole lowland landscape (Somogyi, 2001). Present‐day floodways suffer from several ecological problems originating mainly from channelization, river incision (Borsos & Sendzimir, 2018), and the rapid spread of invasive species accelerated by the abandonment of traditional floodplain use in recent decades (Schindler et al., 2016). River management interventions included cutting off the meanders, creating an artificial river channel, significantly reducing the active floodplain with dykes, and stabilizing the shoreline in some locations.

The characteristic plant community of the narrow waterbank sections along the studied rivers is the softwood gallery forest, whose main tree species are *Salix* spp. (willows) and *Populus* spp. (poplars), belonging to the *Salicaceae* family. The *Salix* genus is represented by native species, and the *Populus* genus is represented by native trees and non‐invasive hybrids of native and non‐native, planted individuals. Poplar plantations (*Populus* x *euramericana*) are common in the region. Native and hybrid poplars generally cannot be clearly distinguished without a genetic survey (Csencsics et al., 2009).

*Acer negundo* (ash‐leaved maple or boxelder), *F*. *pennsylvanica* (green ash), *Robinia pseudoacacia* (black locust), and *Ailanthus altissima* (tree of heaven) are considered to be the most invasive tree species in the temperate zone of Eurasia, the first two of which primarily invade floodplains (Khapugin, 2019). In Hungary, *A*. *negundo* and *F*. *pennsylvanica* pose the greatest threat to softwood forests. *F*. *pennsylvanica* was planted in floodplain habitats starting at the beginning of the 20th century, aiming to promote the transformation of softwood forests into economically more valuable hardwood stands (Csiszár & Bartha, 2004). The spread of *A*. *negundo* was also initiated by planting in the middle of the 20th century, but there is even data on the species from the second half of the 19th century (Udvardy, 2004). Furthermore, the invasive shrub species *Amorpha fruticosa* (false‐indigo bush) is widespread in the Hungarian floodplains, and plantations of invasive *R*. *pseudoacacia* are present in some areas.

**FIGURE 2:**
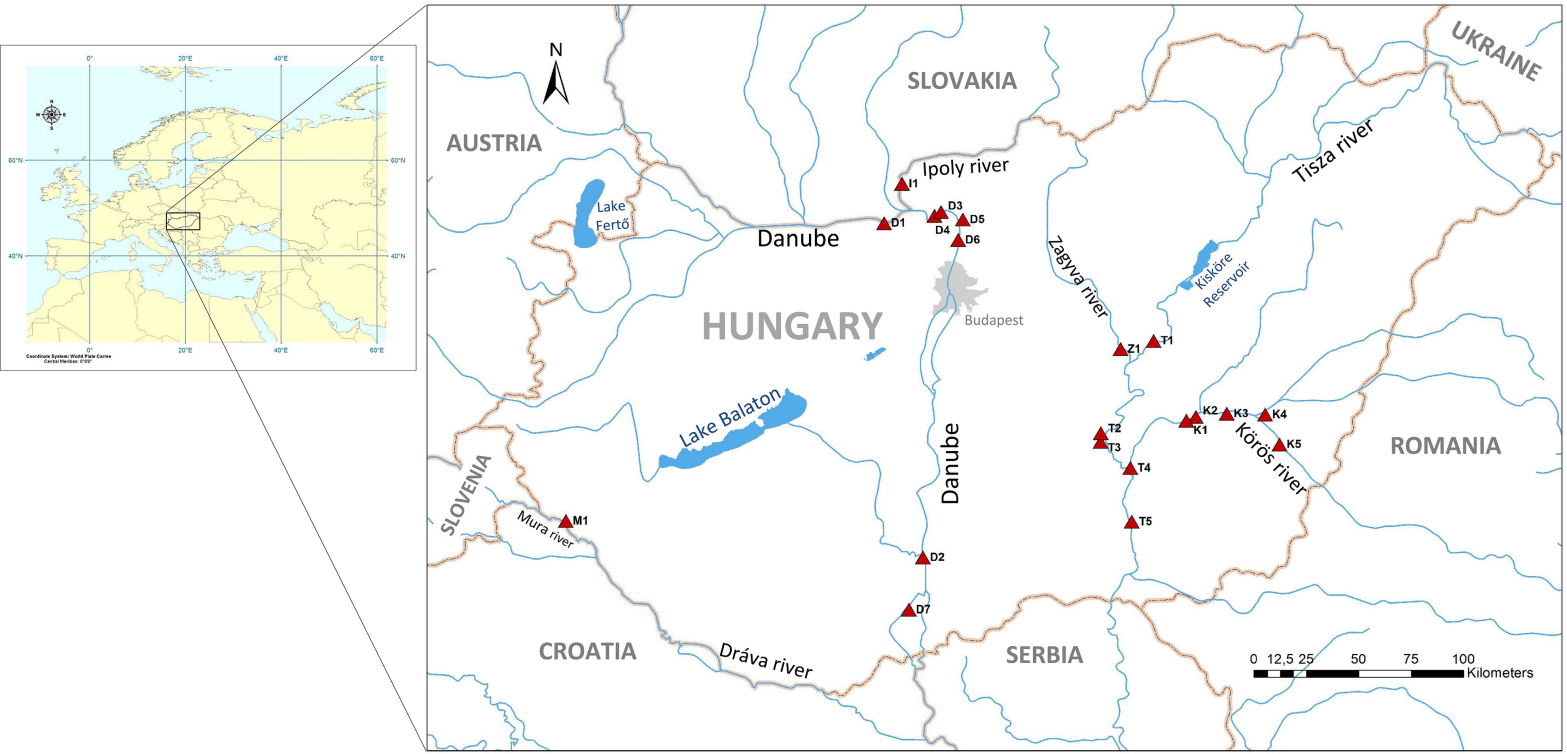
Map of the study sites in Hungary. Sites were located along six rivers: Danube (D1–D7), Mura (M1), Ipoly (I1), Tisza (T1–T5), Zagyva (Z1), and Körös rivers (K1–K5). Source of base maps: ArcGIS 10.1. (ESRI 2012). Main rivers: thin blue lines; national borders: thick brown lines; capital of Hungary: gray; main lakes: blue (Source: Natural Earth; http3: https://www.naturalearthdata.com/downloads/)

For the purpose of examining the woody plant supply and its utilization by beaver (*C*. *fiber*), we selected 20 study sites affected by the spread of invasive species. The sites were located on the active floodplains of the Danube, Mura, Ipoly, Tisza, Zagyva, and Körös rivers (Danube River Basin, Figure 2). Of these, the river regulations affected the Tisza and Körös rivers the most; the Tisza was shortened by 453 km (32%; Somogyi, 2001). Among the studied rivers, the Danube has the greatest average discharge (6745 m³/s at its mouth and 2311 m³/s at Budapest), followed by the Tisza, Mura, Körös, Ipoly, and Zagyva with average discharges of 920, 176, 116, 17, and 17 m³/s at their mouths, respectively (http2: https://www.riversnetwork.org/).

The spontaneous return of the beaver to Hungary began in the early 1990s, followed by a reintroduction program between 1996 and 2008 (Juhász et al., 2019). Regular and comprehensive monitoring results based on systematically collected field data are not available. Based on the limited amount of data, the population size was estimated at 4000–5000 individuals in 2016, predicting that the potential carrying capacity of the habitats in the country is 14,600–18,300 specimens (Čanády et al., 2016).

Along each river, preliminary fieldwork was done and recommendations by nature conservationists and local experts were considered during the study site selection. For more detailed information see also Juhász et al., 2020. All the sites matched the following selection criteria: (1) Traces of intensive fresh beaver activity are clearly visible along the waterbank over a distance of 300 m or more; (2) *A*. *negundo* and/or *F*. *pennsylvanica* occur along the waterbank in softwood gallery forests or narrow waterside softwood groves; and (3) the cover of woody vegetation is continuous along the waterbank over a length of at least 500 m.

During our analyses, we focused primarily on the four most frequently occurring taxa (*Salix* spp., *Populus* spp., *A*. *negundo*, and *F*.* pennsylvanica*). *Salix* spp. were present at all sites, represented by native *Salix alba* and *Salix fragilis* and their hybrid, *Salix* × *rubens*. *Populus* spp. were present at 13 sites, represented by native poplars (*Populus alba*, *P*. × *canescens*, and *P*. × *nigra*), and hybrids of *P*. × *nigra* and *P*. × *euramericana*. *A*. × *negundo* was present at 19 sites, *F*. × *pennsylvanica* at 17 sites. *Salix* spp. and *Populus* spp. were handled at the genus level, because of hybridization, as well as uncertainties in the species‐level identification of the stumps remaining after felling.

### 2.2 Data collection

Each of the study sites was surveyed once, between 2017 and 2020, during a period lasting from the beginning of February until the end of March. At each site, we marked out two 500‐m‐long parallel transects, one directly at the first line of woody species along the waterbank (waterbank transect), and the other 10 m further away (outer transect). Along each transect, we surveyed 50 sampling circles with a 2‐m radius placed 10 m from each other (see also Juhász et al., 2020). At one site, we conducted only a waterbank transect survey, because of the absence of woody plants along the outer transect.

Within the sampling circles, we examined the supply and utilization by beaver of woody plant units available at a height between 0 and 70 cm. Data related to the recruitment layer (preference values obtained in the case of branches with a diameter between 0.8 and 5 cm) were summarized in our earlier publication (Juhász et al., 2020). Based on data collected in parallel at the same study sites, we now focused on the utilization of units (trunks and thick branches) reaching 5 cm in diameter. Data were collected about each unit separately. The diameter was measured using a metal measuring tape. The measurement heights were selected based on our earlier data (Juhász, 2017), where the average utilization height was ~40 cm and the greatest was ~70 cm. Thus, in the case of units branching up to a height of 40 cm, we registered the diameter at a height of 40 cm, while for units branching over a height of 40 cm, the diameter was measured at the branching point.

**FIGURE 3:**
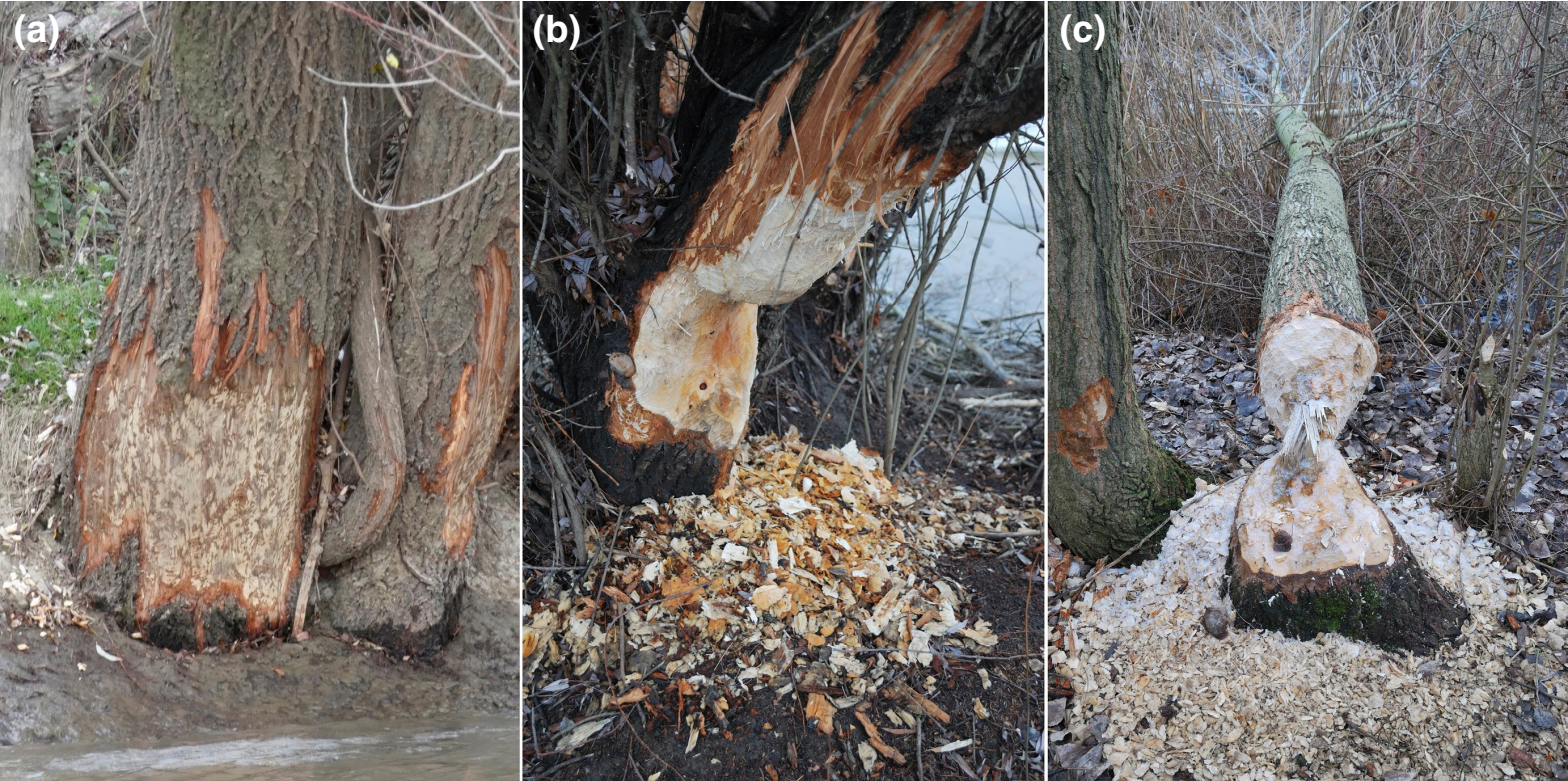
Types of utilization: (a) *debarking* – surface damage to a depth of 0.5–3 cm; (b) *carving* – damage with a depth over 3 cm; and (c) *felling*. Photos: Juhász, E

Utilized units were classified according to three utilization types: *felled*, *carved*, *and debarked* (Figure 3). *Summarized utilization (SU)* refers to the combination of all three utilization types. The term *felled* means that the tree was no longer standing after the beaver's activity. *Carved* trees were those that had been utilized at a depth of at least 3 cm, while those that had only surface damage to a depth between 0.5 and 3 cm were classified as *debarked*. The *greatest carving depth* (GCD) was also registered, which we used to calculate the *carving ratio*: carving ratio = greatest carving depth/diameter. In the case of debarked trees, the GCD was defined as 2 cm, for felled trees the GCD was identical to the diameter of the trunk, while for carved trees we measured the deepest point of carving.

The utilization of a unit was considered “fresh” in the case of a light‐colored surface. By our estimation and based on earlier observations, these beaver signs were at most a few months old, having originated between November and March. We also registered utilized units with a browned surface and an estimated age of up to 2 years, with what we refer to as “old signs” (teeth marks show a sharp contrast on the chewing surface, the trunks are not rotted, and the bark has remained on them). The models presented later were run both for the full dataset (in that fresh and old signs are also considered) and the fresh subset.

Field surveys were always carried out by the same surveyor to avoid analytical problems due to different estimates. We summarized data about 5401 units, taking all twenty study sites together.

### 2.3 Data analysis

The statistical protocol takes a dual approach combining whole‐database models with transect‐level analyses. Because of the similar species composition of different sites studied here, whole‐database models can help to identify the most important factors influencing foraging strategy, avoid over‐explaining rare incidences, and facilitate the collective examination of large quantities of data. However, to detect and interpret potential unique phenomena regarding particular beaver colonies, we present preference data at the transect level, as well. Whole‐database analyses were performed in the R software environment (R Core Team, 2019), while database management and transect‐level calculations were carried out in Excel spreadsheets.

#### 2.3.1 Model selection: the importance of variables and their interactions

We filtered the database to the four most abundant taxa (*Salix* spp., *Populus* spp., *A*. *negundo*, *F*. *pennsylvanica*) present at our study sites. Among units reaching at least 5 cm in diameter, considering the whole dataset, the proportion of all of these four main taxa exceeded 10%, while the proportion of every other taxon was below 1.5%. The proportion of the four main taxa in the supply (relative supply, *p*_i_) was different at each site (waterbank transect: p*_Salix_* = 0.49 ± 0.33 SD, p*_Populus_* = 0.12 ± 0.22 SD, p*_A_*_._ *_negundo_* = 0.12 ± 0.11 SD, p*_F_*_._ *_pennsylvanica_* = 0.24 ± 0.27 SD; outer transect: p*_Salix_* = 0.32 ± 0.30 SD, p*_Populus_* = 0.15 ± 0.23 SD, p*_A_*_._ *_negundo_* = 0.27 ± 0.2 SD, p*_F_*_._ *_pennsylvanica_* = 0.23 ± 0.24 SD; transect‐level data are available in Appendix A).

The importance of the three independent variables (taxon, diameter, and transect) and their paired interactions were calculated in a nested model comparison framework (Appendix B). The three variables and the three interactions were treated as fixed factors, while the site was treated as a random factor (displayed overlined in the equations) in generalized linear mixed models (GLMM) with binomial distribution family using the R package “lme4” (Bates et al., 2015). For each of the importance estimations, two models were compared that differ only in the inclusion/exclusion of the studied fixed factor (Table B1). Four metrics were calculated: likelihood ratio, the significance of the likelihood ratio, the difference in Akaike Information Criterion (Akaike, 1974), and the difference in Bayesian Information Criterion (Schwarz, 1978).

The initial model including interactions is formalized in Equation 1, where Ta stands for taxon, D for diameter, Tr for transect (distance from the water), and S for site.

(1) $$\mathrm{response}\;∼\;\mathrm{Ta}+D+\mathrm{Tr}+\mathrm{Ta}\times D+\mathrm{Ta}\times \mathrm{Tr}+D\times \mathrm{Tr}+\bar{S}$$

This modeling framework was used five times, independently of each other as follows.

- Response variable "summarized utilization" using the full dataset.
- Response variable "summarized utilization" using a subset of the dataset containing only the fresh supply (old signs of utilization with a browned surface were excluded).
- Response variable "felling" using the full dataset.
- Response variable "felling" using a subset of the dataset containing only the fresh supply.
- Response variable "carving ratio" using the full dataset. (The fresh subset was not examined separately because in the case of utilized units with a light surface, it is not possible to determine what proportion of the carving depth is the result of fresh foraging activity, and what proportion was created earlier.)

#### 2.3.2 Factors behind the different utilization of the units

After estimating the importance of variables and their interaction, and finding that interactions are less important than the variables (Table B2), a deeper analysis of the variables was carried out without the interaction terms. GLMM with binomial distribution family was built according to Equation 2.

(2) $$\mathrm{response}\;∼\;\mathrm{Ta}+D+\mathrm{Tr}+\bar{S}$$

Modeling was done using the same response variables as in the case of Equation 1.

#### 2.3.3 Taxon preference

Pairwise comparison of the levels of the "taxon" independent variable was carried out by means of Tukey based on the model defined by Equation 2 using R packages “emmeans” (Lenth, 2020) and “multcomp” (Hothorn et al., 2008).

#### 2.3.4 Diameter selectivity and the type of utilization

We presented the utilization ratio and the percentage of each utilization type within supply groups (combinations of taxon and diameter class). For this, 5 diameter classes were created using the Jenks natural breaks method (5–12; 13–26; 27–46; 47–85; and 86–202 cm) (Jenks, 1967).

For the statistical analysis of the differences in the diameter of felled, carved, debarked, and intact trees, GLMM with Gaussian distribution family was built according to Equation 3, using 8 subsets of the data by the Ta × Tr interaction. This means that the relation between the type of utilization and the diameter was treated separately for each taxon and both transects. In these models, C stands for category (felled, carved, debarked, and intact).

(3) $$\mathrm{diameter}∼C+\bar{S}$$

A pairwise comparison of the levels of the "category" independent variable was carried out by means of Tukey based on the model defined by Equation 3.

#### 2.3.5 Testing the distance‐selectivity relation according to the optimal foraging strategy

Along the transects, we determined the taxon diversity among the units in the supply (*H*_supply_) and among the utilized units (*H*_utilized_, for summarized utilization) using the Shannon diversity index (Shannon, 1948). Within all study sites, *H*_supply_ – *H*_utilized_ differences were compared between the two transects to test the effect of distance on the magnitude of selectivity.

While Shannon diversity provides us with information about taxon selectivity, diameter selectivity was studied using the standard deviation of the diameter. The standard deviation value was calculated for both the supply (SD_d_supply_) and the utilized units (SD_d_utilized_, summarized utilization), and subjected to the same comparisons. The pairwise difference values were compared using the paired *t*‐test for both the Shannon index and standard deviation, after using the Shapiro–Wilk test (Shapiro & Wilk, 1965) for examining normality. For this, pairwise data of those sites were considered, where at least two units were utilized along both transects.

#### 2.3.6 Preferences at the transect level

The number of available and felled units and the number of utilized units were summarized separately for all transects. During this analysis, fresh and old signs of utilization were treated together. Preference was examined using the Bonferroni *Z* test following the Chi‐square goodness of fit test (Neu et al., 1974), and quantified by the Jacobs selectivity index (Jacobs, 1974). Positive values of Jacobs selectivity index (0 < D_i_ ≤ 1) indicate a preference, while negative values (−1 ≤ D_i_ < 0) indicate avoidance. The procedure was performed for summarized utilization and for felling, as well. Pairwise values (summarized utilization and felling) obtained in this way were compared qualitatively with each other and with the results gained from the generalized linear mixed models. This technique helped us to understand and interpret the advantages and limitations of the different methods.

## 3 RESULTS

### 3.1 Factors behind the different utilization of the units

When fresh and old signs of utilization were treated together (full dataset models), the effect of taxon, diameter, and transect all proved to be significant at a level of α = 0.001 for each response variable (summarized utilization, felling, and carving ratio; Table 1). Different taxa were utilized with different ratios, and thin units were preferred to larger ones. In addition, there was a higher ratio of utilization along the waterbank transect than along the outer transect, so foraging intensity was lower 10 m from the water than directly along the waterbank. Models using a subset of the dataset containing only the fresh supply (dealing only with the winter—early spring foraging decisions) did not reveal different tendencies at all. They gave the same outcome as the full dataset models (Table C1).

**TABLE 1:** Results of generalized linear mixed models without interaction terms (defined by Equation 2), using the full dataset (old signs of utilization with a browned surface were included)

	Estimate	Standard error	*z* value	*p* value
Summarized utilization: full dataset				
(Intercept)	0.465	0.191	2.436	.015
taxon_An	−3.140	0.205	−15.348	<.001
taxon_Fp	−2.138	0.148	−14.478	<.001
taxon_P	0.778	0.163	4.760	<.001
diameter	−0.050	0.004	−13.988	<.001
transect_OT	−1.43	0.123	−11.657	<.001
Felling: full dataset				
(Intercept)	0.936	0.215	4.349	<.001
taxon_An	−3.080	0.216	−14.267	<.001
taxon_Fp	−2.009	0.163	−12.354	<.001
taxon_P	0.741	0.188	3.948	<.001
diameter	−0.117	0.006	−18.274	<.001
transect_OT	−1.333	0.134	−9.919	<.001
Carving ratio: full dataset				
(Intercept)	0.942	0.206	4.568	<.001
taxon_An	−3.055	0.213	−14.377	<.001
taxon_Fp	−2.003	0.160	−12.485	<.001
taxon_P	0.733	0.185	3.960	<.001
diameter	−0.113	0.006	−18.276	<.001
transect_OT	−1.328	0.133	−9.993	<.001

Note

The reference level of taxon and transect categorical variables were *Salix* spp. and waterbank transect, respectively. Key: taxon_An—*Acer negundo*, taxon_Fp—*Fraxinus pennsylvanica*, taxon_P—*Populus* spp., transect_OT—outer transect.

Based on the importance of variables determined by the likelihood ratio, in the case of summarized utilization, foraging decisions were mostly explained by taxon, which was followed by the importance of diameter, then that of the transect. In contrast, in the case of felling and carving ratio, the diameter was of slightly higher importance than the taxon (Table B2, full dataset models).

### 3.2 Taxon preference

The order of preference for the most abundant four taxa was the following in the case of all response variables: *Populus* spp. > *Salix* spp. > *F*. *pennsylvanica* > *A*. *negundo*. According to the multiple comparisons of means using the models of the full datasets, pairwise differences in utilization were always significant at a significance level of *α* = 0.001 (Table 2). The beaver preferred softwoods against invasive hardwood species. The results were similar in the case of fresh subset models, only the pairwise differences in the utilization of *A*. *negundo* and *F*. *pennsylvanica* were not significant (Table C2).

**TABLE 2:** Pairwise differences in the utilization of the most abundant four taxa, according to the multiple comparisons of means, based on generalized linear mixed models without interaction terms (defined by Equation 2)

	Estimate	Standard error	z value	p value
Taxa	Summarized utilization: full dataset			
An‐S	−3.140	0.205	−15.348	<.001
Fp‐S	−2.138	0.148	−14.478	<.001
P‐S	0.778	0.164	4.760	<.001
Fp‐An	1.002	0.205	4.887	<.001
P‐An	3.918	0.234	16.696	<.001
P‐Fp	2.916	0.179	16.291	<.001
	Felling: full dataset			
An‐S	−3.080	0.216	−14.267	<.001
Fp‐S	−2.009	0.163	−12.354	<.001
P‐S	0.741	0.188	3.948	<.001
Fp‐An	1.071	0.213	5.020	<.001
P‐An	3.821	0.249	15.346	<.001
P‐Fp	2.750	0.193	14.251	<.001
	Carving ratio: full dataset			
An‐S	−3.055	0.213	−14.377	<.001
Fp‐S	−2.003	0.160	−12.485	<.001
P‐S	0.733	0.185	3.960	<.001
Fp‐An	1.052	0.211	4.978	<.001
P‐An	3.789	0.246	15.413	<.001
P‐Fp	2.737	0.191	14.318	<.001

Note

Full dataset models were used (old signs of utilization with a browned surface were included). Key for the taxa column: S—*Salix* spp., P—*Populus* spp., An—*A*. *negundo*, Fp—*F*. *pennsylvanica*.

### 3.3 Diameter selectivity and the type of utilization

**FIGURE 4:**
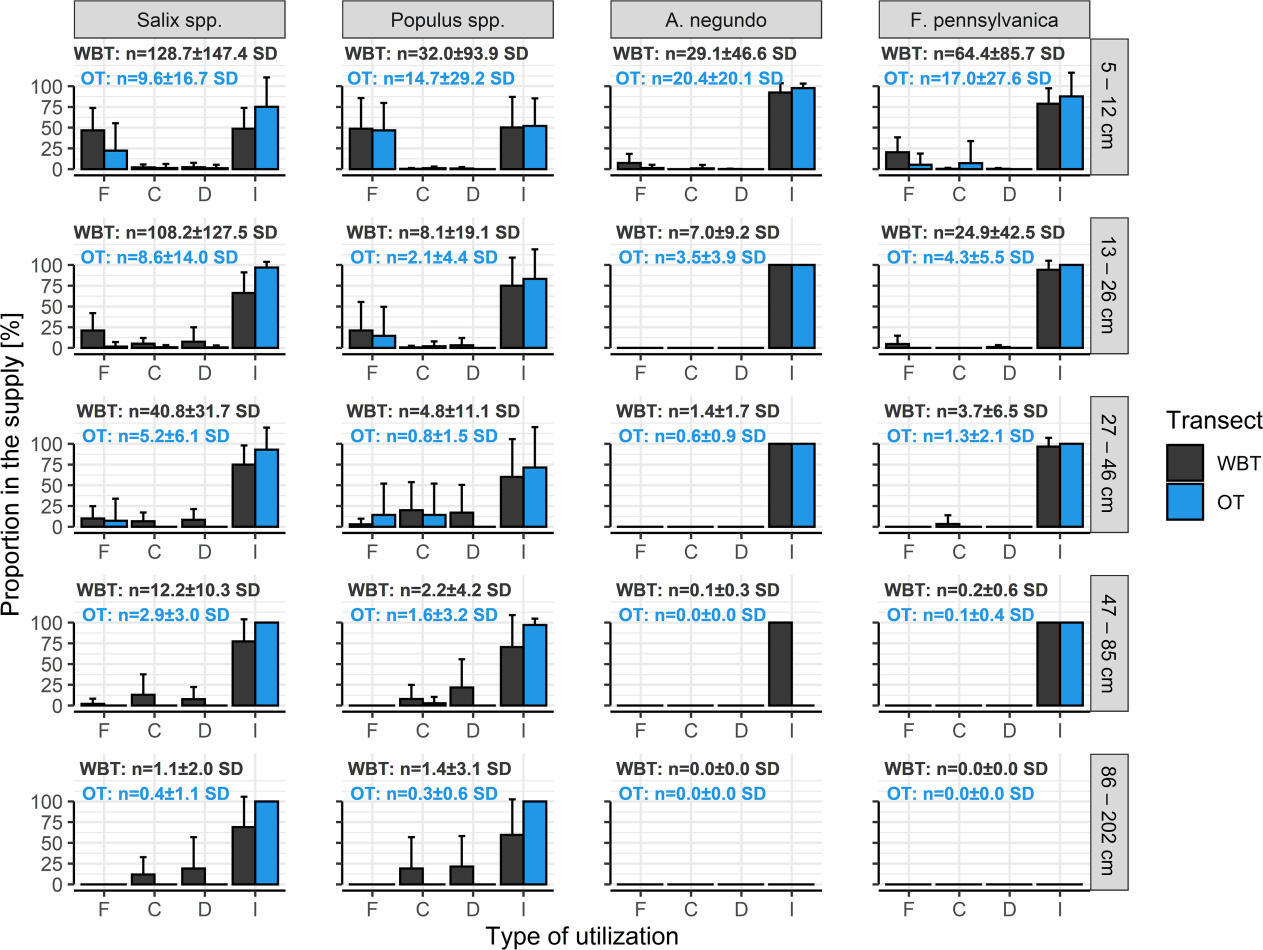
Percentage of each utilization type within the supply groups (combinations of taxon and diameter class). Whiskers display the standard deviation of transect‐level values. Key: F—Felled, C—Carved, D—Debarked, I—Intact, WBT—waterbank transect, OT—outer transect, n—average number of units of the given taxon in the diameter class

The beaver utilized the smallest diameter class (5–12 cm) primarily for *F*. *pennsylvanica* and exclusively for *A*. *negundo* (Figure 4). In contrast to softwood species, the beaver almost never utilized thicker specimens of invasive hardwood species. Taxon and diameter influenced not only the intensity but also the type of utilization. In the greater diameter classes, the rate of carving and debarking increased compared to felling. We thus found that, when the felling and carving ratio were studied, among the independent variables it was a diameter that stood out as being the most important (Table B2). Moreover, it can be seen that units belonging to larger diameter classes were rare along the outer transect, and in parallel with this, debarking occurred there only occasionally.

**FIGURE 5:**
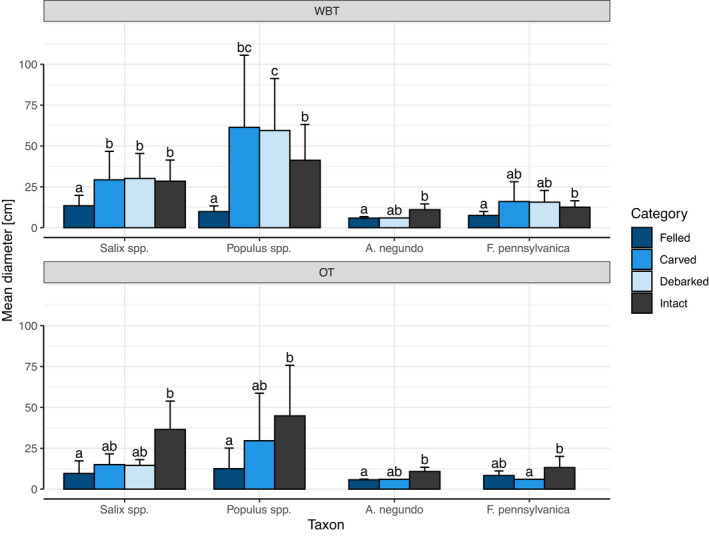
The mean diameter of felled, carved, debarked and intact units in the case of the most abundant four taxa. Whiskers display the standard deviation of transect‐level values. Significance groups (i.e., letters) were generated by means of Tukey according to the GLMM defined in Equation 2, where two groups sharing no common letter(s) are significantly different at level α = 0.05. Key: WBT—waterbank transect, OT—outer transect

Based on models according to Equation 3, differences in the diameter of felled and intact units proved to be significant except in the case of the *F*. *pennsylvanica*—outer transect subset of data. On the other hand, the difference between the mean diameters of these two categories (felled and intact) was higher in the case of *Salix* and *Populus* spp. than that of invasive species (Figure 5). This could be related to the fact that the mean diameter in the supply of the main four taxa was also different (waterbank transect: *d_Salix_* = 25.4 ± 12.05 SD; *d_Populus_* = 42.05 ± 23.8 SD; *d_A_*_._ *_negundo_* = 10.8 ± 3.32 SD; *d_F_*_._ *_pennsylvanica_* = 11.81 ± 3.64; outer transect: *d_Salix_* = 33.82 ± 18.14 SD; *d_Populus_* = 39.21 ± 32.74 SD; *d_A_*_._ *_negundo_* = 10.72 ± 2.58 SD; *d_F_*_._ *_pennsylvanica_* = 13.0 ± 6.4 SD). Comparing the diameter of debarked and carved units to that of felled units, significant differences were found in the case of *Salix* and *Populus* spp. along the waterbank transect. This difference was not statistically supported for invasive species.

### 3.4 Testing the distance‐selectivity relation according to the optimal foraging strategy

The difference in taxon Shannon diversity between supply and utilized units was typically larger along the outer transect than along the waterbank transect at the same site (Table 3). A similar result was obtained in the case of standard deviation for diameter. The difference proved to be significant for both taxon Shannon diversity (−4.028, *df* = 10, *p* < .01) and the diameter's standard deviation (−4.658, *df* = 10, *p* < .001), which indicates that the beaver's selectivity was stronger at a greater distance from the water.

**TABLE 3:** Differences in the Shannon index value and the standard deviation for diameter between the supply units and the utilized units (summarized utilization), calculated for sites where at least two units were utilized along the outer transect

Site	Shannon diversity index for taxa			Standard deviation for diameter		
	WBT: H_supply_ –H_utilized_	OT: H_supply_ ‐H_utilized_	WBT – OT difference	WBT: H_supply_ ‐H_utilized_	OT: H_supply_ ‐H_utilized_	WBT – OT difference
D1	0.401	1.128	−0.727	−1.874	11.844	−13.719
D2	0.401	N/A	N/A	−3.115	N/A	N/A
D3	0.023	0.082	−0.059	5.721	10.055	−4.334
D4	0.370	0.681	−0.311	−0.133	17.718	−17.851
D5	0.256	0.053	0.203	5.547	11.585	−6.038
D6	0.465	N/A	N/A	6.225	N/A	N/A
D7	0.373	N/A	N/A	0.710	N/A	N/A
M1	0.011	0.321	−0.310	−1.523	22.917	−24.44
I1	0.947	N/A	N/A	−4.966	N/A	N/A
T1	0.035	N/A	N/A	0.658	N/A	N/A
T2	0.062	N/A	N/A	−9.759	N/A	N/A
T3	0.305	N/A	N/A	−0.169	N/A	N/A
T4	0.312	N/A	N/A	1.388	N/A	N/A
T5	0.452	1.146	−0.694	−0.044	−2.458	2.502
Z1	0.201	0.609	−0.408	−1.475	19.030	−20.505
K1	0.149	0.584	−0.435	2.114	18.822	−16.708
K2	0.263	0.665	−0.402	−5.151	1.163	−6.314
K3	0.218	N/A	N/A	0.650	N/A	N/A
K4	−0.242	0.036	−0.278	−9.734	6.908	−16.642
K5	0.581	0.719	−0.138	17.175	21.842	−4.667

Abbreviations: WBT, waterbank transect; OT, outer transect.

### 3.5 Preferences at the transect level

**FIGURE 6:**
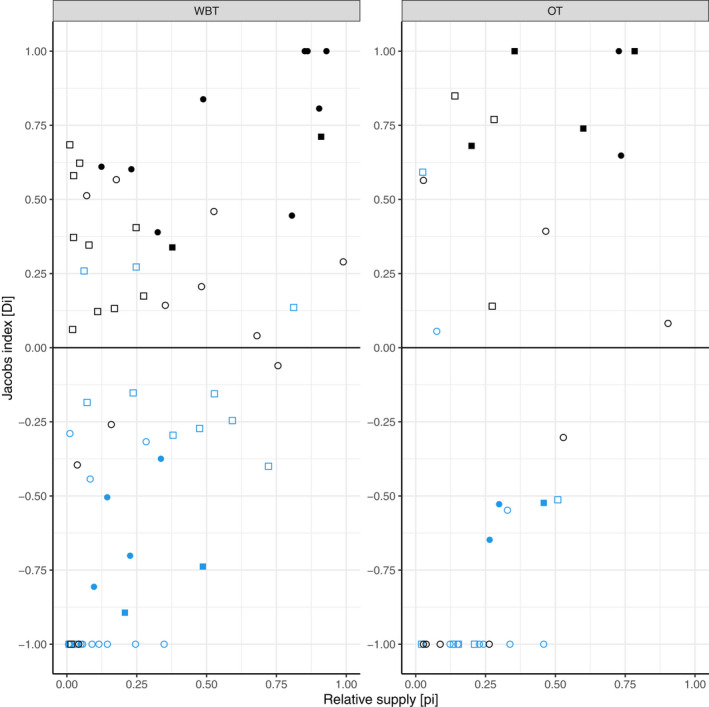
Relation of the Jacobs selectivity index (D_i_) and relative supply (p_i_) calculated for the most abundant four taxa. (Relative supply means the proportion of a given taxon in the total supply.) Values were considered for transects where at least two units were utilized. Key: black circle—*Salix* spp., black square—*Populus* spp., blue circle—*A*. *negundo*, blue square—*F*. *pennsylvanica*, WBT—waterbank transect, OT—outer transect, filled marker—significant Jacobs index value, empty marker—nonsignificant Jacobs index value. Significance level: *α* = 0.05

At 7 sites, the utilization of units with a diameter reaching 5 cm was confined exclusively to the waterbank transect, and at 2 other sites only one unit was utilized along the outer transect. Thus, 20 waterbank transects and 11 outer transects were included in the transect‐level analysis. Jacobs selectivity index values calculated for the same taxon at different sites showed considerable differences along the waterbank transect (Figure D1), as well as along the outer transect (Figure D2). However, the significant results of the Bonferroni *Z* test for summarized utilization showed the preference for *Salix* spp. and *Populus* spp. and avoidance of *A*. *negundo* and *F*. *pennsylvanica* (Figure 6). There were no outstanding results at the transect level.

There could be marked differences in utilization among taxa and also among diameter classes (see Section 3.3). When we considered only felling as utilization, it shaped the preference values considerably, and sometimes it caused remarkable differences even in the results of the Bonferroni *Z* test (Figures D1, D2). For example, in the case of the D2 site's waterbank transect, *Salix* spp. were significantly preferred based on summarized utilization, but in terms of felling a non‐significant avoidance was shown. In parallel, along the T2 site's waterbank transect, a significant preference for *F*. *pennsylvanica* was indicated by felling with a value of D_Fp, felling_ = 1 (cf. D_Fp, summarized utilization_ = 0.136) because only this taxon was felled there, while the others were carved or debarked.

Based on the summarized utilization, *Salix* spp. were significantly preferred along 9 of the 20 waterbank transects (45%) and 2 of the 19 outer transects (11.53%), while *Populus* spp. were significantly preferred along 2 waterbank transects (10%) and 4 outer transects (21.05%). In parallel with this, according to the same method, *A*. *negundo* was significantly avoided along 4 waterbank transects (20%) and 2 outer transects (10.53%), while *F*. *pennsylvanica* was significantly avoided along 2 waterbank transects (10%) and one outer transect (5.26%). Along the D5 site's outer transect, a non‐significant avoidance of *Salix* spp. was found as a result of the high ratio utilization of another taxon, which was rare in the supply (*Cornus sanguinea*, D = 0.743, n.s.) (Appendix A). *Amorpha fruticosa*, the invasive shrub species were utilized along 4 waterbank transects (20%), but no significant preference value was obtained for the species. Three more invasive species, *Acer saccharinum*, *Celtis occidentalis*, and *R*. *pseudoacacia*, were present at certain sites, but these were not utilized by beaver.

The list of other species present in the supply is as follows: *C*. *sanguinea*, *Crataegus monogyna*, *Fraxinus angustifolia* ssp. *pannonica*, *Morus alba*, *Prunus cerasifera*, *Prunus spinosa*, *Rosa* sp., *Ulmus laevi*s, *Ulmus minor*, *Vitis* sp. Among them, only *C*. *sanguinea*, *C*. *monogyna*, and *U*. *laevis* were utilized, without significant preference or avoidance. Jacobs index values calculated for all studied taxa in the supply are presented in detail in Appendix A.

## 4 DISCUSSION

### 4.1 Factors behind the different utilization of the units

Taxon, diameter, and distance from water all significantly influenced the foraging decisions of the beaver. Besides the difference in preference among the taxa, we also found that the beaver avoided large trees and that foraging intensity decreased at a greater distance from the waterbank. These findings are consistent with those of several previous studies (Deardorff & Gorchov, 2021; Jackowiak et al., 2020; Jenkins, 1980).

Diameter selectivity and the avoidance of greater distances can be explained by the energetic costs and the risk of predation (Belovsky, 1984; Salandre et al., 2017). Handling time increases exponentially with the diameter (Fryxell & Doucet, 1993). In the case of old trees, significant extra work could be required not only for felling but also for processing and sectioning the thick branch systems (Jenkins, 1980). Mahoney and Stella (2020) stated that diameter is a more important variable in foraging decisions than taxon, while Jackowiak et al. (2020) found the opposite. Our results suggest that different interpretations of utilization may influence the order of importance among the variables: in terms of felling, the diameter was the most important variable at our study sites, while taxon was the most important factor in summarized utilization (felling, carving, and debarking together). However, we assume that a general rule applicable to all beaver habitats cannot be established, because the order of preference can be influenced by differences in species composition and by the diameter class distribution.

### 4.2 Taxon preference

In our study sites, the beaver preferred softwood (*Salix* and *Populus*) species to the invasive *A*. *negundo* and *F*. *pennsylvanica*, according to the H1 hypothesis. This was also observed in the recruitment layer: softwoods were usually preferred and never significantly avoided, *F*. *pennsylvanica* was significantly preferred at one site, but the invasive species were usually avoided (Juhász et al., 2020).

As beaver do not build dams on the studied rivers, it can be assumed that the trunks and branches are mostly used for feeding purposes, and we interpret our results with this in mind. *Fraxinus* species may have special importance in the beaver's diet, and utilization of the species by beaver has been proven in several studies (Fustec et al., 2001; Nolet et al., 1994; Vorel et al., 2015). In North America, the beaver prefers *F*. *pennsylvanica* to *A*. *negundo* (Dieter & McCabe, 1989). *Acer negundo* was found to be avoided both in its native, North American distribution range (Brzyski & Schulte, 2009), and in Europe (Fustec & Cormier, 2007). Sometimes *A*. *negundo* is considered a preferred species, for example, as second in rank after *Salix* spp. (Poland, Jackowiak et al., 2020), and first in order of preference in North America (Ohio State, Deardorff & Gorchov, 2021). The latter case can be explained especially by the vastly different woody plant supply and the presence of several other less consumed species in that. These noticeable differences point out that the preference values should always be interpreted in the light of species composition.

Nutrients and secondary metabolites of the available food items can influence the foraging decisions of beavers (Bailey et al., 2004; Doucet & Fryxell, 1993). The concentration of inorganic elements in the bark (Tharakan et al., 2003), the sugar content (Kurek et al., 2019), and secondary metabolite composition (Barrales‐Cureño et al., 2020; Palo, 1984; Qazi et al., 2018) can differ among the examined plant taxa. To improve understanding of the experienced order of preference, the effects of these chemicals on digestibility should be examined comparatively in the future. Furthermore, there are remarkable differences in the dry wood density and hardness of different tree species (http4: https://www.wood‐database.com/), which may influence the handling time and should also be considered.

### 4.3 Diameter selectivity and the type of utilization

Certain tree species produce certain metabolites in different proportions at different plant ages (that is, in trees with different diameters) (Wam et al., 2017), which may influence the beaver's diameter selectivity and its differences within the taxa (Basey et al., 1988). However, in the case of all four main taxa, the utilization ratio was the highest within the smallest diameter class (5–12 cm).

Invasive species were almost completely spared the effects of beaver activity after reaching a certain thickness (>13 cm). At the same time, the beaver also utilized the large trunks of the preferred taxa, so the H2 hypothesis was confirmed. Among the diameter classes examined here, the smallest seemed to be the most profitable in terms of felling at both distances from water. The H3 hypothesis was only partially supported: as we expected, larger trunks tended to be debarked or carved and were only rarely felled, but contrary to our expectations, felling was the most frequent utilization type not just among the preferred taxa, but also among the non‐preferred ones. The higher frequency of debarking and carving among the larger trunks is presumably due to the high processing time required for felling them. If alongside the felling of trees, we also consider debarking and carving, which require less energy from the beaver, then the effect exerted on softwood trees was shown to be much greater.

### 4.4 Testing the distance‐selectivity relation according to the optimal foraging strategy

Further from the water, the beaver was significantly more selective in terms of species and diameter than along the waterbank. This increased selectivity supports the optimal and central‐place foraging strategy hypothesis (Belovsky, 1984; Fryxell & Doucet, 1991; Salandre et al., 2017) and thus the H4 hypothesis of this paper. Along the outer transect, the majority of the utilized units belonged to the smallest diameter class (5–12 cm). Mature softwood trees were less affected by beaver activity at greater distances from the waterbank.

While the diameter selection close to the central place depends especially on the energy per handling time ratio, at greater distances the most profitable are the largest trees that can be pulled to the water without sectioning (Gallant et al., 2004). It is more worthwhile for the beaver to fell and pull away smaller trunks to obtain a greater quantity of the resource in less time.

### 4.5 Lessons learned from the transect‐level analysis

It was previously confirmed that the beaver's selective foraging can show general patterns within and among local populations (Vorel et al., 2015). Nevertheless, when it comes to nature conservation issues, special attention should be paid to the possibility of unique phenomena. Due to the differences in utilization among the taxa and/or the low summarized utilization ratio, the transect‐level analysis did not always produce significant results, although the softwood preference tendency was obvious, and no outstanding preference value was gained. It should be noted, however, that at one study site (D5), along the outer transect the beaver selected a native species (*Cornus sanguinea*) that was rare in the supply. This phenomenon is known in the international literature, where it is explained by the need for complementary nutrients which are available in different quantities in certain taxa (Nolet et al., 1994).

Some papers distinguish only between living and felled trees (e.g. Donkor & Fryxell, 1999; Haarberg & Rosell, 2006; Mahoney & Stella, 2020; Vorel et al., 2015) during the analysis of selective foraging. The transect‐level analysis presented here showed that the diameter dependency of the type of utilization may pose a methodological problem. If the diameter‐class distributions of the different taxa are not the same, and only felling is regarded as utilization, then the preference values may shift. For this reason, we recommend that similar studies of preferences should not only consider felling as utilization but also should concentrate on the summarized utilization or on the different types of utilization (felling, debarking, carving).

## 5 CONSERVATION CONSEQUENCES

We predict that the proportionally higher utilization of softwood species will lead to a decrease in their ratio in the canopy layer, while the invasive hardwoods will be released from the beaver's foraging impact after reaching a diameter threshold (~13 cm). Since invasive species are less affected than softwoods, even in the smallest diameter category, they are more likely to reach this critical size. Old softwood trees are constantly exposed to beaver disturbance, in particular to debarking and carving, while larger trunks of invasive hardwood species remain intact. This finding supports the enemy release hypothesis (Keane & Crawley, 2002). The reduction in the quantity of softwoods in the canopy layer, meanwhile, can exert a significant effect on the whole floodplain forest community (Ónodi & Winkler, 2016).

The effects of the beaver's selective foraging on woody plant invasion and the mechanisms of these effects may differ depending on the beaver's order of preference and on the competitive hierarchy of the species that are present, as well as on other local conditions affecting this hierarchy. In eastern Montana, Lesica and Miles (2004) found that the accelerated invasion of *Elaeagnus* and *Tamarix* is driven by preferential utilization of *Populus* spp. and by the higher growth rate of the invasive species along the beaver‐created sunny corridors and canopy gaps. On the other hand, the beaver did not accelerate the exchange of native species for *Elaeagnu*s in a study conducted by Barela and Frey (2016), where the invasive species were utilized in higher proportions.

Based on the results of this article, we can make predictions about the changes happening in the canopy layer, but further, long‐term research is needed to examine the regeneration potential and sprouting ability of trees in European softwood gallery forests threatened by invasive species. Beaver foraging strongly influences canopy closure and forest structure, especially in the proximity of the central place (Mahoney & Stella, 2020). The opening of canopy gaps enhances sapling survival, more rapid growth, and strengthening of branches, and thus the rejuvenation of the forest (Tinya et al., 2020). Due to beaver utilization, larger softwood trunks may be destroyed and replaced, or may undergo substantial morphological changes, turning “bushy” as a consequence of many new offshoots (Johnston & Naiman, 1990; Jones et al., 2009). Our results about the beaver's foraging strategy in this environment should be integrated with knowledge about the plants’ responses to beaver‐made wounds and other factors influencing the competitiveness of softwood and invasive hardwood species.

## 6 CONCLUSIONS

Owing to the higher utilization of preferred softwood taxa, the beaver may accelerate the shift in canopy composition toward invasive species. This is due to the fact that the decline in softwoods is faster because invasive hardwoods are utilized in smaller numbers and almost exclusively at younger ages. At the same time, thin branches can gain strength in beaver‐made forest gaps. Therefore, in the presence of intense natural beaver disturbance, special attention should be paid to ensuring that the native vegetation of these vulnerable floodplain habitats is maintained and that steps are taken to foster its ability to regenerate.

Because the beaver utilizes fewer and mainly thin trees at a distance of 10 m from the water, the protection of large active floodplains could support the coexistence of beaver‐altered softwood stands and mature softwood stands free of the species’ impact. Physiognomically distinct forms of riparian forest patches (altered to varying extents by beaver disturbance) can improve habitat heterogeneity.

The beaver's systematic foraging decisions can lead the succession in predictable directions, which we can better understand if we have sufficient knowledge about the other processes shaping the landscape. Describing and forecasting the effects of beaver activity seem essential for the appropriate planning and implementation of necessary interventions targeting floodplain habitat conservation and reconstruction.

## CONFLICT OF INTEREST

The authors have no conflicts of interest to declare.

## AUTHOR CONTRIBUTIONS

**Erika Juhász:** Conceptualization (equal); Data curation (lead); Formal analysis (equal); Investigation (lead); Methodology (equal); Visualization (lead); Writing – original draft (lead). **Ákos Bede‐Fazekas:** Formal analysis (equal); Methodology (equal); Visualization (supporting); Writing – original draft (equal). **Krisztián Katona:** Formal analysis (supporting); Methodology (supporting); Writing – original draft (equal). **Zsolt Molnár:** Writing – original draft (equal). **Marianna Biró:** Conceptualization (equal); Supervision (lead); Writing – original draft (equal).

## Supporting information

Supplementary Material

Supplementary Material

## Data Availability

The authors confirm that the data supporting the findings of this study are openly available (Juhász et al., 2022).
