# Supplementary material for: Foraging decisions with conservation consequences: Interaction between beavers and invasive tree species
Source: Ecol Evol. 2022 May 15;12(5):e8899. doi: 10.1002/ece3.8899 (PMC9108316; doi:10.1002/ece3.8899)
Supplement: Supplementary file 1 — Supplementary Material [file ECE3-12-e8899-s001.pdf]

Appendix A

Jacobs selectivity index values calculated for all studied taxa in the supply of units thicker than 5 cm in diameter

The following data are shown for all species on transect level: relative supply (p), Jacobs selectivity index value (D), relative utilization (r), result of Bonferroni Z test for frequency (frequent/rare), and result of Bonferroni Z test for preference (preferred/avoided).

Preference values calculated for those OTs where only one unit was utilized are starred.

			Salix spp.					Populus spp.				
Site	Transect	Type of utilization	p	D	r	frequent/rare	preferred/avoided	p	D	r	frequent/rare	preferred/avoided
D1	WBT	summarized	0,4817518	0,20594	0,58537	frequent	ns	0,0146	-1	0	rare	
D1	WBT	felling	0,4817518	0,06673	0,51515	frequent	ns	0,0146	-1	0	rare	
D1	OT	summarized	0,2631579	-1	0	ns		0,14035	0,84906	0,66667	ns	ns
D1	OT	felling	0,2631579	-1	0	ns		0,14035	0,7193	0,5	ns	ns
D2	WBT	summarized	0,1237113	0,61028	0,36842	rare	preferred	0,01031	0,68421	0,05263	rare	ns
D2	WBT	felling	0,1237113	-0,0608	0,11111	frequent	ns	0,01031	-1	0	rare	
D2	OT	summarized	0,2643678			ns						
D2	OT	felling	0,2643678			ns						
D3	WBT	summarized	0,9892473	0,28958	0,99405	frequent	ns					
D3	WBT	felling	0,9892473	0,2459	0,99346	frequent	ns					
D3	OT	summarized	0,9032258	0,08197	0,91667	frequent	ns					
D3	OT	felling	0,9032258	1	1	frequent	preferred					
D4	WBT	summarized	0,8514493	1	1	frequent	preferred					
D4	WBT	felling	0,8514493	1	1	frequent	preferred					
D4	OT	summarized	0,7272727	1	1	frequent	preferred					
D4	OT	felling	0,7272727	1	1	frequent	preferred					
D5	WBT	summarized	0,9031008	0,80648	0,98864	frequent	preferred					
D5	WBT	felling	0,9031008	0,79131	0,98765	frequent	preferred					
D5	OT	summarized	0,5285714	-0,3028	0,375	frequent	ns					
D5	OT	felling	0,5285714	-0,7412	0,14286	frequent	avoided					
D6	WBT	summarized	0,8616601	1	1	frequent	preferred					
D6	WBT	felling	0,8616601	1	1	frequent	preferred					
D6	OT	summarized	0,6590909			frequent						
D6	OT	felling	0,6590909			frequent						
D7	WBT	summarized	0,8055556	0,44551	0,91525	frequent	preferred					
D7	WBT	felling	0,8055556	0,35982	0,89796	frequent	ns					
D7	OT	summarized	0,2923077	1	1	ns	preferred*					
D7	OT	felling	0,2923077	1	1	ns	preferred*					
M1	WBT	summarized	0,6804124	0,04027	0,69767	frequent	ns	0,02062	0,06145	0,02326	rare	ns
M1	WBT	felling	0,6804124	-0,0313	0,66667	frequent	ns	0,02062	-1	0	rare	
M1	OT	summarized	0,7352941	0,64789	0,92857	frequent	preferred					
M1	OT	felling	0,7352941	1	1	frequent	preferred					
I1	WBT	summarized	0,488	0,83781	0,91525	frequent	preferred	0,024	0,58031	0,08475	rare	ns
I1	WBT	felling	0,488	1	1	frequent	preferred	0,024	-1	0	rare	
I1	OT	summarized						0,00813			rare	
I1	OT	felling						0,00813			rare	
T1	WBT	summarized	0,7555556	-0,0608	0,73239	frequent	ns	0,17037	0,13208	0,21127	ns	ns
T1	WBT	felling	0,7555556	0,00682	0,75806	frequent	ns	0,17037	0,02453	0,17742	ns	ns
T1	OT	summarized	0,3382353			ns		0				
T1	OT	felling	0,3382353			ns		0				
T2	WBT	summarized	0,1588235	-0,2591	0,1	rare	ns	0,02353	0,3719	0,05	rare	ns
T2	WBT	felling	0,1588235	-1	0	rare		0,02353	-1	0	rare	
T2	OT	summarized	0,0246914			rare		0,03704			rare	
T2	OT	felling	0,0246914			rare		0,03704			rare	
T3	WBT	summarized	0,9291339	1	1	frequent	preferred					
T3	WBT	felling	0,9291339	1	1	frequent	preferred					
T4	WBT	summarized	0,037037	-0,3953	0,01639	rare	ns	0,91005	0,71141	0,98361	frequent	preferred
T4	WBT	felling	0,037037	-0,3882	0,01667	rare	ns	0,91005	0,70723	0,98333	frequent	preferred
T4	OT	summarized	0,0531915	-1	0	rare		0,08511	-1	0	rare	
T4	OT	felling	0,0531915	-1	0	rare		0,08511	-1	0	rare	
T5	WBT	summarized	0,3522013	0,14291	0,42029	frequent	ns	0,37736	0,33831	0,55072	frequent	preferred
T5	WBT	felling	0,3522013	-0,0278	0,33962	frequent	ns	0,37736	0,46273	0,62264	frequent	preferred
T5	OT	summarized	0,0277778	-1	0	rare		0,35417	1	1	frequent	preferred
T5	OT	felling	0,0277778	-1	0	rare		0,35417	1	1	frequent	preferred
Z1	WBT	summarized	0,325	0,38926	0,52273	ns	preferred	0,11	0,12185	0,13636	rare	ns
Z1	WBT	felling	0,325	0,35	0,5	ns	ns	0,11	0,14839	0,14286	rare	ns
Z1	OT	summarized	0,0877193	-1	0	rare		0,2807	0,76978	0,75	ns	ns
Z1	OT	felling	0,0877193	-1	0	rare		0,2807	0,76978	0,75	ns	ns
K1	WBT	summarized	0,0422535	-1	0	rare		0,27465	0,17427	0,35	frequent	ns
K1	WBT	felling	0,0422535	-1	0	rare		0,27465	0,22619	0,375	frequent	ns
K1	OT	summarized	0,0375	-1	0	rare		0,6	0,73913	0,90909	frequent	preferred
K1	OT	felling	0,0375	-1	0	rare		0,6	0,71429	0,9	frequent	preferred
K2	WBT	summarized	0,1769912	0,56679	0,4375	ns	ns	0,24779	0,40496	0,4375	ns	ns
K2	WBT	felling	0,1769912	-0,2648	0,11111	ns	ns	0,24779	0,71717	0,66667	ns	preferred
K2	OT	summarized						0,78378	1	1	frequent	preferred
K2	OT	felling						0,78378	1	1	frequent	preferred
K3	WBT	summarized	0,2307692	0,60183	0,54688	ns	preferred					
K3	WBT	felling	0,2307692	0,53846	0,5	ns	preferred					
K3	OT	summarized	0,0222222			rare						
K3	OT	felling	0,0222222			rare						
K4	WBT	summarized	0,0708861	0,51271	0,19149	rare	ns	0,04557	0,62238	0,17021	rare	ns
K4	WBT	felling	0,0708861	0,2721	0,11765	rare	ns	0,04557	-1	0	rare	ns
K4	OT	summarized	0,0277778	0,56425	0,09302	rare	ns	0,2	0,68067	0,56818	ns	preferred
K4	OT	felling	0,0275862	0,55801	0,09091	rare	ns	0,2	0,70492	0,59091	ns	preferred
K5	WBT	summarized	0,5263158	0,45946	0,75	frequent	ns	0,07895	0,34615	0,15	rare	ns
K5	WBT	felling	0,5263158	0,4012	0,72222	frequent	ns	0,07895	0,4	0,16667	rare	ns
K5	OT	summarized	0,4657534	0,39286	0,66667	frequent	ns	0,27397	0,13978	0,33333	ns	ns
K5	OT	felling	0,4657534	0,06849	0,5	frequent	ns	0,27397	0,45205	0,5	ns	ns

			Acer negundo					Fraxinus pennsylvanica				
Site	Transect	Type of utilization	p	D	r	frequent/rare	preferred/avoided	p	D	r	frequent/rare	preferred/avoided
D1	WBT	summarized	0,22628	-0,7016	0,04878	ns	avoided	0,24818	0,27213	0,36585	frequent	ns
D1	WBT	felling	0,22628	-0,6385	0,06061	ns	avoided	0,24818	0,38123	0,42424	frequent	ns
D1	OT	summarized	0,22807	-1	0	ns		0,21053	-1	0	ns	
D1	OT	felling	0,22807	-1	0	ns		0,21053	-1	0	ns	
D2	WBT	summarized	0,14433	-0,5045	0,05263	rare	avoided	0,72165	-0,4	0,52632	frequent	ns
D2	WBT	felling	0,14433	-0,1487	0,11111	rare	ns	0,72165	0,14894	0,77778	rare	ns
D2	OT	summarized	0,27586			ns		0,45977				ns
D2	OT	felling	0,27586			ns		0,45977				ns
D3	WBT	summarized	0,01075	-0,2896	0,00595	rare	ns					
D3	WBT	felling	0,01075	-0,2459	0,00654	rare	ns					
D3	OT	summarized	0,07527	0,05521	0,08333	rare	ns	0,02151	-1	0	rare	
D3	OT	felling	0,07527	-1	0	rare		0,02151	-1	0	rare	
D4	WBT	summarized	0,14493	-1	0	rare						
D4	WBT	felling	0,14493	-1	0	rare						
D4	OT	summarized	0,24242	-1	0	ns						
D4	OT	felling	0,24242	-1	0	ns						
D5	WBT	summarized	0,0969	-0,8065	0,01136	rare	avoided					
D5	WBT	felling	0,0969	-0,7913	0,01235	rare	avoided					
D5	OT	summarized	0,32857	-0,5481	0,125	ns	ns					
D5	OT	felling	0,32857	-0,1005	0,28571	ns	ns					
D6	WBT	summarized	0,11462	-1	0	rare						
D6	WBT	felling	0,11462	-1	0	rare						
D6	OT	summarized	0,29545			ns		0,02273			rare	
D6	OT	felling	0,29545			ns		0,02273			rare	
D7	WBT	summarized	0,08333	-0,443	0,0339	rare	ns	0,07222	-0,1847	0,05085	rare	ns
D7	WBT	felling	0,08333	-0,3623	0,04082	rare	ns	0,07222	-0,0883	0,06122	rare	ns
D7	OT	summarized	0,13846	-1	0	ns		0,46154	-1	0	frequent	
D7	OT	felling	0,13846	-1	0	ns		0,46154	-1	0	frequent	
M1	WBT	summarized	0,01031	-1	0	rare		0,23711	-0,1525	0,18605	ns	ns
M1	WBT	felling	0,01031	-1	0	rare		0,23711	0,10613	0,27778	ns	ns
M1	OT	summarized	0,26471	-0,6479	0,07143	rare	avoided					
M1	OT	felling	0,26471	-1	0	rare						
I1	WBT	summarized	0,348	-1	0	frequent						
I1	WBT	felling	0,348	-1	0	frequent						
I1	OT	summarized	0,43089			rare						
I1	OT	felling	0,43089			rare						
T1	WBT	summarized	0,00741	-1	0	rare		0,00741	-1	0	rare	
T1	WBT	felling	0,00741	-1	0	rare		0,00741	-1	0	rare	
T1	OT	summarized	0,16176			ns		0,42647			frequent	
T1	OT	felling	0,16176			ns		0,42647			frequent	
T2	WBT	summarized	0,00588	-1	0	rare		0,81176	0,1357	0,85	frequent	ns
T2	WBT	felling	0,00588	-1	0	rare		0,81176	1	1	frequent	preferred
T2	OT	summarized	0,11111			ns		0,76543			frequent	
T2	OT	felling	0,11111			ns		0,76543			frequent	
T3	WBT	summarized						0,0315	-1	0	rare	
T3	WBT	felling						0,0315	-1	0	rare	
T4	WBT	summarized	0,03704	-1	0	rare		0,01587	-1	0	rare	
T4	WBT	felling	0,03704	-1	0	rare		0,01587	-1	0	rare	
T4	OT	summarized	0,44681	-1	0	frequent		0,3617	1	1	frequent	preferred*
T4	OT	felling	0,44681	-1	0	frequent		0,3617	1	1	frequent	preferred*
T5	WBT	summarized	0,05031	-1	0	rare		0,20755	-0,8937	0,01449	ns	avoided
T5	WBT	felling	0,05031	-1	0	rare		0,20755	-0,8632	0,01887	ns	avoided
T5	OT	summarized	0,45833	-1	0	frequent		0,15278	-1	0	ns	
T5	OT	felling	0,45833	-1	0	frequent		0,15278	-1	0	ns	
Z1	WBT	summarized	0,09	-1	0	rare		0,475	-0,2725	0,34091	frequent	ns
Z1	WBT	felling	0,09	-1	0	rare		0,475	-0,2391	0,35714	frequent	ns
Z1	OT	summarized	0,12281	-1	0	rare		0,50877	-0,513	0,25	frequent	ns
Z1	OT	felling	0,12281	-1	0	rare		0,50877	-0,513	0,25	frequent	ns
K1	WBT	summarized	0,05634	-1	0	rare		0,52817	-0,1555	0,45	frequent	ns
K1	WBT	felling	0,05634	-1	0	rare		0,52817	-0,1801	0,4375	frequent	ns
K1	OT	summarized	0,3375	-1	0	ns		0,025	0,59184	0,09091	rare	ns
K1	OT	felling	0,3375	-1	0	ns		0,025	0,625	0,1	rare	ns
K2	WBT	summarized	0,00885	-1	0	rare		0,48673	-0,7381	0,125	frequent	avoided
K2	WBT	felling	0,00885	-1	0	rare		0,48673	-0,5369	0,22222	frequent	ns
K2	OT	summarized						0,13514	-1	0	rare	
K2	OT	felling						0,13514	-1	0	rare	
K3	WBT	summarized	0,33654	-0,3746	0,1875	frequent	avoided	0,37981	-0,2951	0,25	frequent	ns
K3	WBT	felling	0,33654	-0,3295	0,2037	frequent	ns	0,37981	-0,2285	0,27778	frequent	ns
K3	OT	summarized	0,91111			frequent		0,06667			rare	
K3	OT	felling	0,91111			frequent		0,06667			rare	
K4	WBT	summarized	0,28354	-0,3172	0,17021	frequent	ns	0,59241	-0,2457	0,46809	frequent	ns
K4	WBT	felling	0,28354	-0,1252	0,23529	frequent	ns	0,59241	0,11559	0,64706	frequent	ns
K4	OT	summarized	0,29861	-0,5278	0,11628	frequent	avoided	0,45833	-0,5234	0,2093	frequent	avoided
K4	OT	felling	0,29655	-0,5336	0,11364	frequent	avoided	0,45517	-0,5798	0,18182	frequent	avoided
K5	WBT	summarized	0,24561	-1	0	ns		0,0614	0,25882	0,1	rare	ns
K5	WBT	felling	0,24561	-1	0	ns		0,0614	0,31288	0,11111	rare	ns
K5	OT	summarized	0,15068	-1	0	ns						
K5	OT	felling	0,15068	-1	0	ns						

			Amorpha fruticosa					Acer saccharinum				
Site	Transect	Type of utilization	p	D	r	frequent/rare	preferred/avoided	p	D	r	frequent/rare	preferred/avoided
D1	WBT	summarized										
D1	WBT	felling										
D1	OT	summarized										
D1	OT	felling										
D2	WBT	summarized										
D2	WBT	felling										
D2	OT	summarized										
D2	OT	felling										
D3	WBT	summarized										
D3	WBT	felling										
D3	OT	summarized										
D3	OT	felling										
D4	WBT	summarized										
D4	WBT	felling										
D4	OT	summarized										
D4	OT	felling										
D5	WBT	summarized										
D5	WBT	felling										
D5	OT	summarized										
D5	OT	felling										
D6	WBT	summarized						0,02372	-1	0	rare	
D6	WBT	felling						0,02372	-1	0	rare	
D6	OT	summarized						0,01136			rare	
D6	OT	felling						0,01136			rare	
D7	WBT	summarized										
D7	WBT	felling										
D7	OT	summarized										
D7	OT	felling										
M1	WBT	summarized										
M1	WBT	felling										
M1	OT	summarized										
M1	OT	felling										
I1	WBT	summarized										
I1	WBT	felling										
I1	OT	summarized										
I1	OT	felling										
T1	WBT	summarized	0,05926	-0,0268	0,05634	rare	ns					
T1	WBT	felling	0,05926	0,04527	0,06452	rare	ns					
T1	OT	summarized	0,07353			rare						
T1	OT	felling	0,07353			rare						
T2	WBT	summarized										
T2	WBT	felling										
T2	OT	summarized	0,04938			rare						
T2	OT	felling	0,04938			rare						
T3	WBT	summarized	0,03937	-1	0	rare						
T3	WBT	felling	0,03937	-1	0	rare						
T4	WBT	summarized										
T4	WBT	felling										
T4	OT	summarized										
T4	OT	felling										
T5	WBT	summarized	0,01258	0,07167	0,01449	rare	ns					
T5	WBT	felling	0,01258	0,20307	0,01887	rare	ns					
T5	OT	summarized	0,00694	-1	0	rare						
T5	OT	felling	0,00694	-1	0	rare						
Z1	WBT	summarized										
Z1	WBT	felling										
Z1	OT	summarized										
Z1	OT	felling										
K1	WBT	summarized	0,05634	0,49442	0,15	rare	ns					
K1	WBT	felling	0,05634	0,58893	0,1875	rare	ns					
K1	OT	summarized										
K1	OT	felling										
K2	WBT	summarized	0,07965	-1	0	rare						
K2	WBT	felling	0,07965	-1	0	rare						
K2	OT	summarized	0,08108	-1	0	rare						
K2	OT	felling	0,08108	-1	0	rare						
K3	WBT	summarized	0,03365	-0,3738	0,01563	rare	ns					
K3	WBT	felling	0,03365	-0,2972	0,01852	rare	ns					
K3	OT	summarized										
K3	OT	felling										
K4	WBT	summarized	0,00759	-1	0	rare						
K4	WBT	felling	0,00759	-1	0	rare						
K4	OT	summarized	0,01389	-1	0	rare						
K4	OT	felling	0,01389	-1	0	rare						
K5	WBT	summarized	0,07018	-1	0	rare						
K5	WBT	felling	0,07018	-1	0	rare						
K5	OT	summarized										
K5	OT	felling										

			Celtis occidentalis					Cornus sanguinea				
Site	Transect	Type of utilization	p	D	r	frequent/rare	preferred/avoided	p	D	r	frequent/rare	preferred/avoided
D1	WBT	summarized										
D1	WBT	felling										
D1	OT	summarized						0,01754	-1	0	rare	
D1	OT	felling						0,01754	-1	0	rare	
D2	WBT	summarized										
D2	WBT	felling										
D2	OT	summarized										
D2	OT	felling										
D3	WBT	summarized										
D3	WBT	felling										
D3	OT	summarized										
D3	OT	felling										
D4	WBT	summarized										
D4	WBT	felling										
D4	OT	summarized										
D4	OT	felling										
D5	WBT	summarized										
D5	WBT	felling										
D5	OT	summarized						0,12857	0,74286	0,5	rare	ns
D5	OT	felling						0,12857	0,80074	0,57143	rare	ns
D6	WBT	summarized										
D6	WBT	felling										
D6	OT	summarized										
D6	OT	felling										
D7	WBT	summarized						0,02778	-1	0	rare	
D7	WBT	felling						0,02778	-1	0	rare	
D7	OT	summarized						0,01538	-1	0	rare	
D7	OT	felling						0,01538	-1	0	rare	
M1	WBT	summarized										
M1	WBT	felling										
M1	OT	summarized										
M1	OT	felling										
I1	WBT	summarized						0,012	-1	0	rare	
I1	WBT	felling						0,012	-1	0	rare	
I1	OT	summarized										
I1	OT	felling										
T1	WBT	summarized										
T1	WBT	felling										
T1	OT	summarized										
T1	OT	felling										
T2	WBT	summarized										
T2	WBT	felling										
T2	OT	summarized										
T2	OT	felling										
T3	WBT	summarized										
T3	WBT	felling										
T4	WBT	summarized										
T4	WBT	felling										
T4	OT	summarized										
T4	OT	felling										
T5	WBT	summarized										
T5	WBT	felling										
T5	OT	summarized										
T5	OT	felling										
Z1	WBT	summarized										
Z1	WBT	felling										
Z1	OT	summarized										
Z1	OT	felling										
K1	WBT	summarized										
K1	WBT	felling										
K1	OT	summarized										
K1	OT	felling										
K2	WBT	summarized										
K2	WBT	felling										
K2	OT	summarized										
K2	OT	felling										
K3	WBT	summarized										
K3	WBT	felling										
K3	OT	summarized										
K3	OT	felling										
K4	WBT	summarized										
K4	WBT	felling										
K4	OT	summarized										
K4	OT	felling										
K5	WBT	summarized										
K5	WBT	felling										
K5	OT	summarized	0,0137	-1	0	rare						
K5	OT	felling	0,0137	-1	0	rare						

			Crataegus monogyna					Fraxinus angustifolia ssp. pannonica				
Site	Transect	Type of utilization	p	D	r	frequent/rare	preferred/avoided	p	D	r	frequent/rare	preferred/avoided
D1	WBT	summarized	0,0073	-1	0	rare						
D1	WBT	felling	0,0073	-1	0	rare						
D1	OT	summarized	0,08772	0,67742	0,33333	ns	ns					
D1	OT	felling	0,08772	0,82456	0,5	ns	ns					
D2	WBT	summarized										
D2	WBT	felling										
D2	OT	summarized										
D2	OT	felling										
D3	WBT	summarized										
D3	WBT	felling										
D3	OT	summarized										
D3	OT	felling										
D4	WBT	summarized										
D4	WBT	felling										
D4	OT	summarized										
D4	OT	felling										
D5	WBT	summarized										
D5	WBT	felling										
D5	OT	summarized										
D5	OT	felling										
D6	WBT	summarized										
D6	WBT	felling										
D6	OT	summarized										
D6	OT	felling										
D7	WBT	summarized										
D7	WBT	felling										
D7	OT	summarized										
D7	OT	felling										
M1	WBT	summarized										
M1	WBT	felling										
M1	OT	summarized										
M1	OT	felling										
I1	WBT	summarized										
I1	WBT	felling										
I1	OT	summarized	0,06504			rare						
I1	OT	felling	0,06504			rare						
T1	WBT	summarized										
T1	WBT	felling										
T1	OT	summarized										
T1	OT	felling										
T2	WBT	summarized										
T2	WBT	felling										
T2	OT	summarized										
T2	OT	felling										
T3	WBT	summarized										
T3	WBT	felling										
T4	WBT	summarized										
T4	WBT	felling										
T4	OT	summarized										
T4	OT	felling										
T5	WBT	summarized										
T5	WBT	felling										
T5	OT	summarized										
T5	OT	felling										
Z1	WBT	summarized										
Z1	WBT	felling										
Z1	OT	summarized										
Z1	OT	felling										
K1	WBT	summarized										
K1	WBT	felling										
K1	OT	summarized										
K1	OT	felling										
K2	WBT	summarized										
K2	WBT	felling										
K2	OT	summarized										
K2	OT	felling										
K3	WBT	summarized										
K3	WBT	felling										
K3	OT	summarized										
K3	OT	felling										
K4	WBT	summarized										
K4	WBT	felling										
K4	OT	summarized						0,0069	0,54011	0,02273	rare	ns
K4	OT	felling						0,0069	0,54011	0,02273	rare	ns
K5	WBT	summarized										
K5	WBT	felling										
K5	OT	summarized										
K5	OT	felling										

			Morus alba					Prunus cerasifera				
Site	Transect	Type of utilization	p	D	r	frequent/rare	preferred/avoided	p	D	r	frequent/rare	preferred/avoided
D1	WBT	summarized	0,0146	-1	0	rare						
D1	WBT	felling	0,0146	-1	0	rare						
D1	OT	summarized										
D1	OT	felling										
D2	WBT	summarized										
D2	WBT	felling										
D2	OT	summarized										
D2	OT	felling										
D3	WBT	summarized										
D3	WBT	felling										
D3	OT	summarized										
D3	OT	felling										
D4	WBT	summarized										
D4	WBT	felling										
D4	OT	summarized	0,0303	-1	0	rare						
D4	OT	felling										
D5	WBT	summarized										
D5	WBT	felling										
D5	OT	summarized	0,01429	-1	0	rare						
D5	OT	felling	0,01429	-1	0	rare						
D6	WBT	summarized										
D6	WBT	felling										
D6	OT	summarized	0,01136			rare						
D6	OT	felling	0,01136			rare						
D7	WBT	summarized										
D7	WBT	felling										
D7	OT	summarized										
D7	OT	felling										
M1	WBT	summarized										
M1	WBT	felling										
M1	OT	summarized										
M1	OT	felling										
I1	WBT	summarized	0,004	-1	0	rare		0,044	-1	0	rare	
I1	WBT	felling	0,004	-1	0	rare		0,044	-1	0	rare	
I1	OT	summarized						0,02439			rare	
I1	OT	felling						0,02439			rare	
T1	WBT	summarized										
T1	WBT	felling										
T1	OT	summarized										
T1	OT	felling										
T2	WBT	summarized										
T2	WBT	felling										
T2	OT	summarized										
T2	OT	felling										
T3	WBT	summarized										
T3	WBT	felling										
T4	WBT	summarized										
T4	WBT	felling										
T4	OT	summarized										
T4	OT	felling										
T5	WBT	summarized										
T5	WBT	felling										
T5	OT	summarized										
T5	OT	felling										
Z1	WBT	summarized										
Z1	WBT	felling										
Z1	OT	summarized										
Z1	OT	felling										
K1	WBT	summarized										
K1	WBT	felling										
K1	OT	summarized										
K1	OT	felling										
K2	WBT	summarized										
K2	WBT	felling										
K2	OT	summarized										
K2	OT	felling										
K3	WBT	summarized										
K3	WBT	felling										
K3	OT	summarized										
K3	OT	felling										
K4	WBT	summarized										
K4	WBT	felling										
K4	OT	summarized										
K4	OT	felling										
K5	WBT	summarized	0,01754	-1	0	rare						
K5	WBT	felling	0,01754	-1	0	rare						
K5	OT	summarized	0,06849	-1	0	ns		0,0137	-1	0	rare	
K5	OT	felling	0,06849	-1	0	ns		0,0137	-1	0	rare	

			Prunus spinosa					Robinia pseudoacacia				
Site	Transect	Type of utilization	p	D	r	frequent/rare	preferred/avoided	p	D	r	frequent/rare	preferred/avoided
D1	WBT	summarized										
D1	WBT	felling										
D1	OT	summarized										
D1	OT	felling										
D2	WBT	summarized										
D2	WBT	felling										
D2	OT	summarized										
D2	OT	felling										
D3	WBT	summarized										
D3	WBT	felling										
D3	OT	summarized										
D3	OT	felling										
D4	WBT	summarized										
D4	WBT	felling										
D4	OT	summarized										
D4	OT	felling										
D5	WBT	summarized										
D5	WBT	felling										
D5	OT	summarized										
D5	OT	felling										
D6	WBT	summarized										
D6	WBT	felling										
D6	OT	summarized										
D6	OT	felling										
D7	WBT	summarized										
D7	WBT	felling										
D7	OT	summarized										
D7	OT	felling										
M1	WBT	summarized										
M1	WBT	felling										
M1	OT	summarized										
M1	OT	felling										
I1	WBT	summarized						0,076	-1	0	rare	
I1	WBT	felling						0,076	-1	0	rare	
I1	OT	summarized	0,02439			rare		0,44715			frequent	
I1	OT	felling	0,02439			rare		0,44715			frequent	
T1	WBT	summarized										
T1	WBT	felling										
T1	OT	summarized										
T1	OT	felling										
T2	WBT	summarized										
T2	WBT	felling										
T2	OT	summarized										
T2	OT	felling										
T3	WBT	summarized										
T3	WBT	felling										
T4	WBT	summarized										
T4	WBT	felling										
T4	OT	summarized										
T4	OT	felling										
T5	WBT	summarized										
T5	WBT	felling										
T5	OT	summarized										
T5	OT	felling										
Z1	WBT	summarized										
Z1	WBT	felling										
Z1	OT	summarized										
Z1	OT	felling										
K1	WBT	summarized										
K1	WBT	felling										
K1	OT	summarized										
K1	OT	felling										
K2	WBT	summarized										
K2	WBT	felling										
K2	OT	summarized										
K2	OT	felling										
K3	WBT	summarized										
K3	WBT	felling										
K3	OT	summarized										
K3	OT	felling										
K4	WBT	summarized										
K4	WBT	felling										
K4	OT	summarized										
K4	OT	felling										
K5	WBT	summarized										
K5	WBT	felling										
K5	OT	summarized										
K5	OT	felling										

Site	Transect	Type of utilization	Rosa sp. p	D	r	frequent/rare	preferred/avoided	Ulmus laevis p	D	r	frequent/rare	preferred/avoided
D1	WBT	summarized										
D1	WBT	felling										
D1	OT	summarized						0,01754	-1	0	rare	
D1	OT	felling						0,01754	-1	0	rare	
D2	WBT	summarized										
D2	WBT	felling										
D2	OT	summarized										
D2	OT	felling										
D3	WBT	summarized										
D3	WBT	felling										
D3	OT	summarized										
D3	OT	felling										
D4	WBT	summarized										
D4	WBT	felling										
D4	OT	summarized										
D4	OT	felling										
D5	WBT	summarized										
D5	WBT	felling										
D5	OT	summarized										
D5	OT	felling										
D6	WBT	summarized										
D6	WBT	felling										
D6	OT	summarized										
D6	OT	felling										
D7	WBT	summarized						0,01111	-1	0	rare	
D7	WBT	felling						0,01111	-1	0	rare	
D7	OT	summarized						0,09231	-1	0	rare	
D7	OT	felling						0,09231	-1	0	rare	
M1	WBT	summarized						0,05155	0,30728	0,09302	rare	ns
M1	WBT	felling						0,05155	0,03955	0,05556	rare	ns
M1	OT	summarized										
M1	OT	felling										
I1	WBT	summarized	0,004	-1	0	rare						
I1	WBT	felling	0,004	-1	0	rare						
I1	OT	summarized										
I1	OT	felling										
T1	WBT	summarized										
T1	WBT	felling										
T1	OT	summarized										
T1	OT	felling										
T2	WBT	summarized										
T2	WBT	felling										
T2	OT	summarized						0,01235			rare	
T2	OT	felling						0,01235			rare	
T3	WBT	summarized										
T3	WBT	felling										
T4	WBT	summarized										
T4	WBT	felling										
T4	OT	summarized										
T4	OT	felling										
T5	WBT	summarized										
T5	WBT	felling										
T5	OT	summarized										
T5	OT	felling										
Z1	WBT	summarized										
Z1	WBT	felling										
Z1	OT	summarized										
Z1	OT	felling										
K1	WBT	summarized						0,02817	0,28972	0,05	rare	ns
K1	WBT	felling						0,02817	-1	0	rare	
K1	OT	summarized										
K1	OT	felling										
K2	WBT	summarized										
K2	WBT	felling										
K2	OT	summarized										
K2	OT	felling										
K3	WBT	summarized						0,00481	-1	0	rare	
K3	WBT	felling						0,00481	-1	0	rare	
K3	OT	summarized										
K3	OT	felling										
K4	WBT	summarized										
K4	WBT	felling										
K4	OT	summarized										
K4	OT	felling										
K5	WBT	summarized										
K5	WBT	felling										
K5	OT	summarized						0,0137	-1	0	rare	
K5	OT	felling						0,0137	-1	0	rare	

Site	Transect	Type of utilization	Ulmus minor					Vitis sp.				
			p	D	r	frequent/rare	preferred/avoided	p	D	r	frequent/rare	preferred/avoided
D1	WBT	summarized	0,0073	-1	0	rare						
D1	WBT	felling	0,0073	-1	0	rare						
D1	OT	summarized						0,03509	-1	0	rare	
D1	OT	felling						0,03509	-1	0	rare	
D2	WBT	summarized										
D2	WBT	felling										
D2	OT	summarized										
D2	OT	felling										
D3	WBT	summarized										
D3	WBT	felling										
D3	OT	summarized										
D3	OT	felling										
D4	WBT	summarized						0,00362	-1	0	rare	
D4	WBT	felling						0,00362	-1	0	rare	
D4	OT	summarized										
D4	OT	felling										
D5	WBT	summarized										
D5	WBT	felling										
D5	OT	summarized										
D5	OT	felling										
D6	WBT	summarized										
D6	WBT	felling										
D6	OT	summarized										
D6	OT	felling										
D7	WBT	summarized										
D7	WBT	felling										
D7	OT	summarized										
D7	OT	felling										
M1	WBT	summarized										
M1	WBT	felling										
M1	OT	summarized										
M1	OT	felling										
I1	WBT	summarized										
I1	WBT	felling										
I1	OT	summarized										
I1	OT	felling										
T1	WBT	summarized										
T1	WBT	felling										
T1	OT	summarized										
T1	OT	felling										
T2	WBT	summarized										
T2	WBT	felling										
T2	OT	summarized										
T2	OT	felling										
T3	WBT	summarized										
T3	WBT	felling										
T4	WBT	summarized										
T4	WBT	felling										
T4	OT	summarized						0,05319	-1	0	rare	
T4	OT	felling						0,05319	-1	0	rare	
T5	WBT	summarized										
T5	WBT	felling										
T5	OT	summarized										
T5	OT	felling										
Z1	WBT	summarized										
Z1	WBT	felling										
Z1	OT	summarized										
Z1	OT	felling										
K1	WBT	summarized						0,01408	-1	0	rare	
K1	WBT	felling						0,01408	-1	0	rare	
K1	OT	summarized										
K1	OT	felling										
K2	WBT	summarized										
K2	WBT	felling										
K2	OT	summarized										
K2	OT	felling										
K3	WBT	summarized						0,01442	-1	0	rare	
K3	WBT	felling						0,01442	-1	0	rare	
K3	OT	summarized										
K3	OT	felling										
K4	WBT	summarized										
K4	WBT	felling										
K4	OT	summarized										
K4	OT	felling										
K5	WBT	summarized										
K5	WBT	felling										
K5	OT	summarized										
K5	OT	felling										
