# Supplementary material for: Foraging decisions with conservation consequences: Interaction between beavers and invasive tree species
Source: Ecol Evol. 2022 May 15;12(5):e8899. doi: 10.1002/ece3.8899 (PMC9108316; doi:10.1002/ece3.8899)
Supplement: Supplementary file 2 — Supplementary Material [file ECE3-12-e8899-s002.pdf]

Supplementary material

Appendix B
Model selection: importance of variables and their interactions

1. Formalization of the initial models including interactions

The importance of the three independent variables (taxon, diameter, transect) and their paired interactions were calculated in a nested model comparison framework detailed below.

$$response \sim Ta + D + Tr + Ta \times D + Ta \times Tr + D \times Tr + \bar{S} \quad \text{(B1)}$$

$$response \sim Ta + D + Tr + Ta \times Tr + D \times Tr + \bar{S} \quad \text{(B2)}$$

$$response \sim Ta + D + Tr + Ta \times D + D \times Tr + \bar{S} \quad \text{(B3)}$$

$$response \sim Ta + D + Tr + Ta \times D + Ta \times Tr + \bar{S} \quad \text{(B4)}$$

$$response \sim Ta + D + Tr + D \times Tr + \bar{S} \quad \text{(B5)}$$

$$response \sim Ta + D + Tr + Ta \times Tr + \bar{S} \quad \text{(B6)}$$

$$response \sim Ta + D + Tr + Ta \times D + \bar{S} \quad \text{(B7)}$$

$$response \sim D + Tr + D \times Tr + \bar{S} \quad \text{(B8)}$$

$$response \sim Ta + Tr + Ta \times Tr + \bar{S} \quad \text{(B9)}$$

$$response \sim Ta + D + Ta \times D + \bar{S} \quad \text{(B10)}$$

Table B1 summarizes the compared models used for estimation of the importance of the fixed factors. The initial model is formalized in Eq. B1, where Ta stands for taxon, D for diameter, Tr for transect, and S for site.

Eq. B2-4 are submodels of Eq. B1, and Eq. B8-10 are also submodels of Eq. B5-7. Random factor is the same for Eq. B1-10. Four metrics were calculated: likelihood ratio, significance of the likelihood ratio, difference in Akaike Information Criterion (AIC; Akaike 1974) and difference in Bayesian Information Criterion (BIC; Schwarz, 1978).

Table B1. Fixed factors and the two models compared for estimation of the importance. Key: Ta – taxon, D – diameter, Tr – transect.

Fixed factor	Model including the fixed factor	Model excluding the fixed factor
Ta×D	Eq. B1	Eq. B2
Ta×Tr	Eq. B1	Eq. B3
D×Tr	Eq. B1	Eq. B4
Ta	Eq. B5	Eq. B8
D	Eq. B6	Eq. B9
Tr	Eq. B7	Eq. B10

This modelling framework was used five times independently of each other for

- response variable "summarized utilization" using the full dataset,
- response variable "summarized utilization " using a subset of the dataset containing only the fresh supply,
- response variable "felling" using the full dataset,
- response variable "felling " using a subset of the dataset containing only the fresh supply,
- response variable "carving ratio" using the full dataset.

2. Results of the initial models including interactions examining the variables’ importance

For all the five response variables, the foraging decision was most explained by taxon and diameter (Table B2). Transect was found to be a less important variable, Interactions were typically far less important factors than the

three main variables, although the importance of taxon x diameter interaction and the main variables were of similar magnitude in the case of summarized utilization (both for the full dataset and the subset of the fresh supply). For all the five response variables, taxon x diameter was found to be the most important interaction, while, the importance of taxon-transect and diameter x transect interactions were similar to each other and their order was not coherent. Whichever variable importance measure out of likelihood ratio, AIC difference and BIC difference is examined, the main findings are similar. The only exception is BIC difference for the two response variables on the subsetted dataset ("summarized utilization: fresh", "felling: fresh"), which measure tends to downweight the importance of taxon x diameter and taxon x transect, respectively.

Table B2 Likelihood ratio, likelihood ratio significance, AIC and BIC values calculated for the fixed factors in the initial models including interactions. Key: Ta – taxon, D – diameter, Tr – transect.

	likelihood ratio	likelihood ratio significance	AIC difference	BIC difference
Summarized utilization: full dataset				
Ta×D	121.218	< 0.001	115.218	95.578
Ta×Tr	24.638	< 0.001	18.638	-1.003
D×Tr	32.571	< 0.001	30.571	24.024
Ta	657.972	< 0.001	651.971	632.331
D	253.315	< 0.001	251.315	244.768
Tr	168.233	< 0.001	166.233	159.686
Felling: full dataset				
Ta×D	67.588	< 0.001	61.588	41.947
Ta×Tr	12.997	0.005	6.997	-12.644
D×Tr	12.560	< 0.001	10.560	4.013
Ta	532.434	< 0.001	526.434	506.793
D	612.268	< 0.001	610.268	603.721
Tr	126.103	< 0.001	124.103	117.556
Carving ratio: full dataset				
Ta×D	69.112	< 0.001	63.112	43.471
Ta×Tr	12.238	0.007	6.238	-13.403
D×Tr	15.466	< 0.001	13.466	6.919
Ta	542.087	< 0.001	536.087	516.446
D	608.255	< 0.001	606.255	599.708
Tr	126.477	< 0.001	124.477	117.930
Summarized utilization: fresh subset				
Ta×D	69.392	< 0.001	63.392	44.158
Ta×Tr	23.337	< 0.001	17.337	-1.897
D×Tr	24.043	< 0.001	22.043	15.632
Ta	288.508	< 0.001	282.508	263.274
D	63.586	< 0.001	61.586	55.175
Tr	63.965	< 0.001	61.965	55.554
Felling: fresh subset				
Ta×D	34.644	< 0.001	28.644	9.410
Ta×Tr	15.060	0.002	9.060	-10.174
D×Tr	11.602	< 0.001	9.602	3.190
Ta	203.624	< 0.001	197.624	178.390
D	268.185	< 0.001	266.185	259.773
Tr	42.239	< 0.001	40.239	33.828

Based on the importance of three main variables (in contrast to the interactions) we conclude that interactions are best removed from the modeling framework, and, hence, the importance of the main variables were studied in this simplified framework.

Appendix C
Foraging decisions in the winter-early spring period: Models, using a subset of the dataset containing only the fresh supply

Table C1 Results of generalized linear mixed models without interaction terms (defined by Eq.2.), using a subset of the dataset containing only the fresh supply. Reference level of taxon and transect categorical variables were *Salix* spp. and waterbank transect, respectively. Key: taxon_An – Acer negundo, taxon_Fp – Fraxinus pennsylvanica, taxon_P – Populus spp., transect_OT – outer transect.

	Estimate	Standard Error	z value	p value
**Summarized utilization: fresh subset**				
(Intercept)	-0.929	0.203	-4.574	< 0.001
taxon_An	-2.427	0.274	-8.856	< 0.001
taxon_Fp	-1.870	0.202	-9.240	< 0.001
taxon_P	1.041	0.195	5.348	< 0.001
diameter	-0.032	0.004	-7.523	< 0.001
transect_OT	-1.175	0.161	-7.283	< 0.001
**Felling: fresh subset**				
(Intercept)	-0.201	0.257	-0.781	0.435
taxon_An	-2.247	0.303	-7.419	< 0.001
taxon_Fp	-1.634	0.240	-6.813	< 0.001
taxon_P	1.173	0.250	4.690	< 0.001
diameter	-0.136	0.012	-11.684	< 0.001
transect_OT	-1.136	0.195	-5.842	< 0.001

Table C2 Pairwise differences in the utilization of the most abundant four taxa, according to the multiple comparision of means, based on generalized linear mixed models without interaction terms (defined by Eq.2.). Full dataset models were used (old signs of utilization with browned surface were included). Key for the taxa column: S – *Salix* spp., P – *Populus* spp., An – *A. negundo*, Fp – *F. pennsylvanica*.

	Estimate	Standard Error	z value	p value
**taxa**	**summarized utilization: fresh subset**			
An-S	-2.427	0.274	-8.856	< 0.001
Fp-S	-1.870	0.202	-9.240	< 0.001
P-S	1.041	0.195	5.348	< 0.001
Fp-An	0.557	0.287	1.939	0.206
P-An	3.468	0.303	11.429	< 0.001
P-Fp	2.911	0.232	12.549	< 0.001
	**felling: fresh subset**			
An-S	-2.247	0.303	-7.419	< 0.001
Fp-S	-1.634	0.240	-6.813	< 0.001
P-S	1.173	0.250	4.690	< 0.001
Fp-An	0.613	0.309	1.985	0.190
P-An	3.420	0.337	10.156	< 0.001
P-Fp	2.807	0.266	10.536	< 0.001

Appendix D Visualisation of the results gained from the site level preference analysis

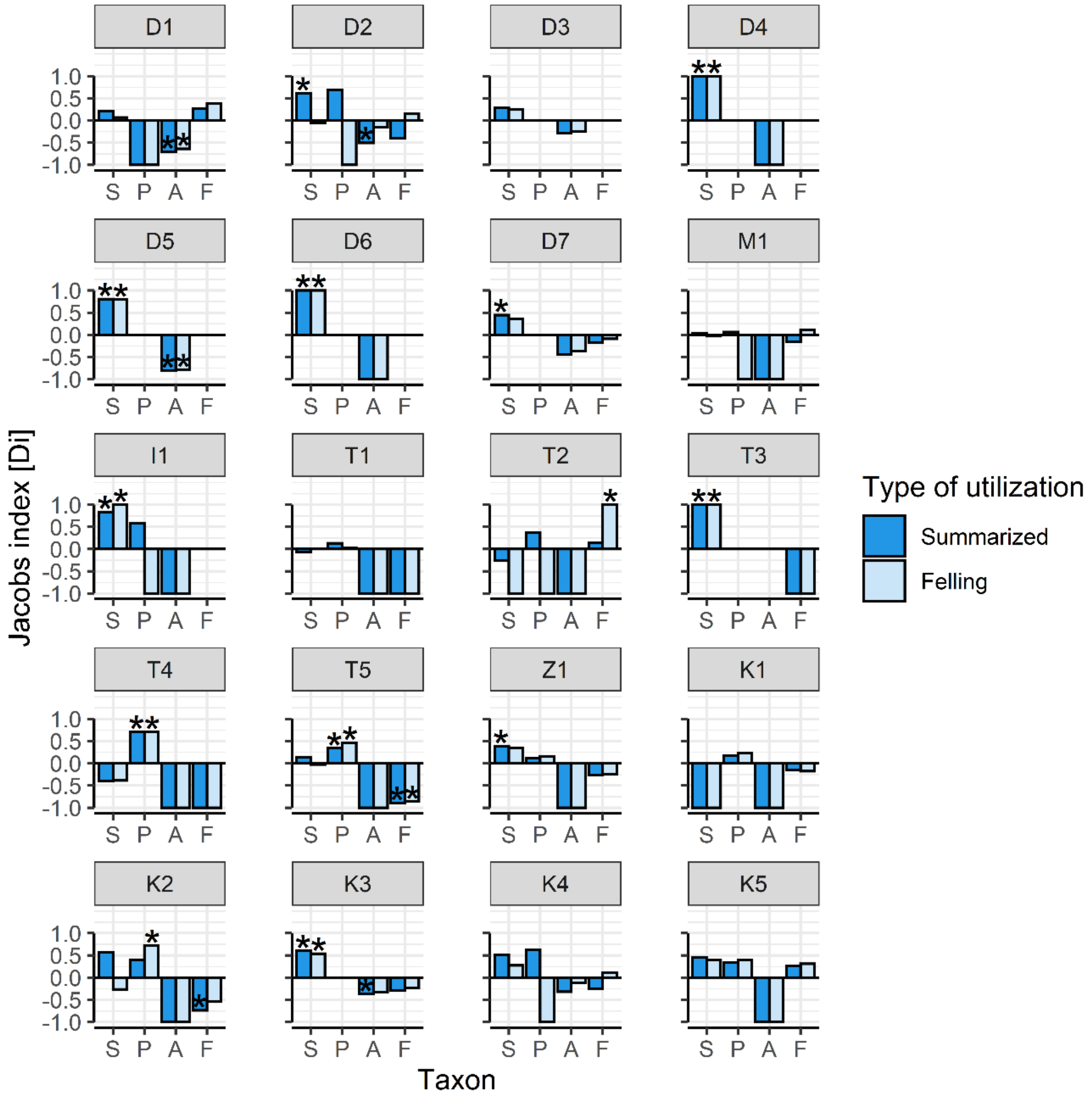

Fig. D1 Jacobs index (D_i) values calculated for the most abundant four taxa along the waterbank transect at each sites. Positive values indicate preference, while negative values mean avoidance. Key: S – *Salix* spp., P – *Populus* spp., A – *Acer negundo*, F – *Fraxinus pennsylvanica*, * – significant preference or avoidance based on Bonferroni Z test (significance level: α=0.05).

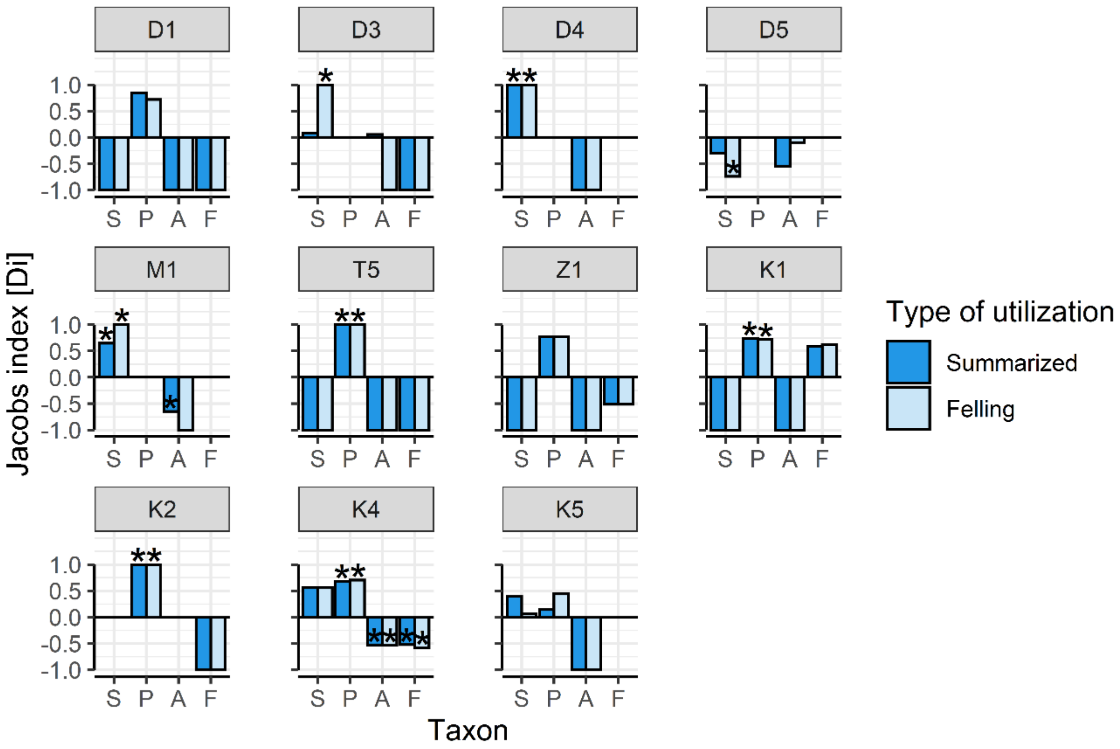

Fig. D2 Jacobs index (D_i) values calculated for the most abundant four taxa along the outer transect at each sites where at least two units were utilized. Positive values indicate preference, while negative values mean avoidance. Key: S – *Salix* spp., P – *Populus* spp., A – *Acer negundo*, F – *Fraxinus pennsylvanica*, * – significant preference or avoidance based on Bonferroni Z test (significance level: α=0.05).
